# Supplementary material for: Patterns of Segmental Strain of the Left Ventricle in Extremely Premature Infants
Source: Pediatr Rep. 2025 Dec 1;17(6):126. doi: 10.3390/pediatric17060126 (PMC12736103; doi:10.3390/pediatric17060126)

Table S1. Correlation between parameters of regional strain and weight, gestation age

Correlation with weight																			
Level		MV						PM						AP					
wall		AntSept	Ant	Lat	Post	Inf	Sept	AntSept	Ant	Lat	Post	Inf	Sept	AntSept	Ant	Lat	Post	Inf	Sept
SL Peak G	R	0.024	0.062	-0.110	0.054	0.135	-0.166	0.017	0.003	-0.038	0.160	0.299	-0.082	0.044	-0.025	-0.050	0.253	0.386	0.018
	p_adj	0.987	0.987	0.987	0.987	0.987	0.987	0.987	0.990	0.987	0.987	0.716	0.987	0.987	0.987	0.987	0.727	0.459	0.987
Time SL Peak G	R	-0.039	0.104	0.024	-0.120	-0.021	0.097	-0.032	0.062	0.067	-0.084	-0.108	-0.022	-0.138	0.161	0.059	0.076	-0.274	0.118
	p_adj	0.987	0.987	0.987	0.987	0.987	0.987	0.987	0.987	0.987	0.987	0.987	0.987	0.987	0.987	0.987	0.987	0.716	0.987
SL Peak S	R	0.049	0.118	-0.093	0.073	0.198	-0.123	0.072	0.014	-0.024	0.168	0.296	-0.021	0.121	-0.048	-0.008	0.278	0.344	0.069
	p_adj	0.987	0.987	0.987	0.987	0.987	0.987	0.987	0.987	0.987	0.987	0.716	0.987	0.987	0.987	0.987	0.716	0.713	0.987
Time SL Peak S	R	-0.144	-0.122	-0.060	-0.097	-0.078	-0.103	-0.155	-0.097	-0.056	-0.048	-0.081	-0.029	-0.208	-0.133	0.043	-0.065	-0.108	0.027
	p_adj	0.987	0.987	0.987	0.987	0.987	0.987	0.987	0.987	0.987	0.987	0.987	0.987	0.987	0.987	0.987	0.987	0.987	0.987
SL Peak P	R	0.135	-0.021	-0.064	0.136	-0.006	-0.013	0.166	-0.040	-0.110	0.169	0.006	0.058	0.159	-0.064	-0.084	0.124	-0.026	0.066
	p_adj	0.987	0.987	0.987	0.987	0.987	0.987	0.987	0.987	0.987	0.987	0.987	0.987	0.987	0.987	0.987	0.987	0.987	0.987
Time SL Peak P	R	0.047	0.218	-0.005	0.008	0.124	0.049	0.085	0.152	-0.028	0.067	0.193	0.112	0.063	0.129	-0.094	0.027	-0.283	0.079
	p_adj	0.987	0.987	0.987	0.987	0.987	0.987	0.987	0.987	0.987	0.987	0.987	0.987	0.987	0.987	0.987	0.987	0.716	0.987
SC Peak G	R	0.006	0.099	-0.128	0.029	-0.002	-0.053	-0.045	-0.085	0.016	0.198	-0.009	-0.092	0.058	0.127	-0.124	-0.307	-0.249	-0.110
	p_adj	0.989	0.961	0.961	0.987	0.997	0.961	0.975	0.961	0.989	0.907	0.989	0.961	0.961	0.961	0.961	0.821	0.821	0.961
Time SC Peak G	R	0.119	-0.137	0.069	0.101	0.119	0.084	0.030	-0.258	-0.125	0.096	-0.056	0.024	-0.109	-0.216	-0.112	0.071	0.216	0.176
	p_adj	0.961	0.961	0.961	0.961	0.961	0.961	0.987	0.821	0.961	0.961	0.961	0.987	0.961	0.821	0.961	0.961	0.821	0.961
SC Peak S	R	-0.005	0.052	-0.212	0.029	0.061	0.015	0.016	0.037	-0.073	0.143	0.103	0.026	0.077	0.003	0.012	-0.145	-0.203	-0.037
	p_adj	0.989	0.961	0.821	0.987	0.961	0.989	0.989	0.987	0.961	0.961	0.961	0.987	0.961	0.997	0.989	0.961	0.901	0.987
Time SC Peak S	R	-0.088	-0.250	-0.100	0.044	-0.076	-0.162	-0.101	-0.132	-0.238	-0.122	-0.241	-0.148	-0.295	-0.021	-0.021	-0.046	-0.114	-0.343
	p_adj	0.961	0.821	0.961	0.975	0.961	0.961	0.961	0.961	0.821	0.961	0.821	0.961	0.821	0.987	0.987	0.975	0.961	0.821
SC Peak P	R	-0.028	0.026	0.173	0.052	-0.167	-0.174	-0.006	0.049	-0.059	0.231	-0.123	-0.051	0.197	0.124	-0.031	-0.264	-0.263	0.045
	p_adj	0.987	0.987	0.961	0.961	0.961	0.961	0.989	0.975	0.961	0.821	0.961	0.968	0.907	0.961	0.987	0.821	0.821	0.975
Time SC Peak P	R	0.012	-0.063	0.039	-0.058	-0.069	-0.064	-0.024	0.288	-0.181	-0.261	-0.023	-0.024	-0.094	0.053	-0.222	-0.256	0.031	0.133
	p_adj	0.989	0.961	0.987	0.961	0.961	0.961	0.987	0.821	0.961	0.821	0.987	0.987	0.961	0.961	0.821	0.821	0.987	0.961
SR Peak G	R	-0.115	-0.173	-0.191	-0.124	0.010	0.037	-0.141	-0.316	-0.388	-0.394	-0.313	-0.105	-0.187	-0.220	-0.166	-0.099	0.006	-0.083
	p_adj	0.961	0.961	0.926	0.961	0.989	0.987	0.961	0.821	0.450	0.450	0.821	0.961	0.961	0.821	0.961	0.961	0.989	0.961
Time SR Peak G	R	-0.054	0.037	0.130	0.103	0.113	-0.008	0.054	0.251	0.291	0.121	-0.021	-0.236	-0.193	-0.123	0.229	-0.093	-0.209	0.079
	p_adj	0.961	0.987	0.961	0.961	0.961	0.989	0.961	0.821	0.821	0.961	0.987	0.821	0.926	0.961	0.821	0.961	0.838	0.961

Correlation with gestation age																			
Level		MV						PM						AP					
wall		AntSept	Ant	Lat	Post	Inf	Sept	AntSept	Ant	Lat	Post	Inf	Sept	AntSept	Ant	Lat	Post	Inf	Sept
SL Peak G	R	-0.011	-0.073	-0.232	-0.018	0.014	-0.222	-0.010	-0.083	-0.247	0.021	0.207	-0.084	-0.022	-0.073	-0.224	0.050	0.296	0.027
	p_adj	0.987	0.987	0.851	0.987	0.987	0.910	0.987	0.987	0.727	0.987	0.987	0.987	0.987	0.987	0.910	0.987	0.716	0.987
Time SL Peak G	R	0.066	0.058	0.078	-0.008	0.023	0.256	0.069	-0.007	0.243	-0.041	-0.061	0.114	-0.103	-0.004	0.207	0.130	-0.210	0.056
	p_adj	0.987	0.987	0.987	0.987	0.987	0.716	0.987	0.987	0.730	0.987	0.987	0.987	0.987	0.987	0.987	0.987	0.987	0.987
SL Peak S	R	0.029	-0.034	-0.186	0.022	0.061	-0.171	0.034	-0.097	-0.180	0.035	0.207	-0.060	0.034	-0.127	-0.146	0.091	0.288	0.040
	p_adj	0.987	0.987	0.987	0.987	0.987	0.987	0.987	0.987	0.987	0.987	0.987	0.987	0.987	0.987	0.987	0.987	0.716	0.987
Time SL Peak S	R	-0.088	-0.111	-0.027	-0.060	-0.123	-0.073	-0.084	-0.087	0.002	0.010	-0.121	-0.036	-0.156	-0.099	-0.023	0.033	-0.061	0.141
	p_adj	0.987	0.987	0.987	0.987	0.987	0.987	0.987	0.987	0.990	0.987	0.987	0.987	0.987	0.987	0.987	0.987	0.987	0.987
SL Peak P	R	0.101	0.011	-0.099	0.121	0.027	-0.034	0.101	-0.015	-0.195	0.086	0.016	0.034	0.077	-0.049	-0.256	-0.011	-0.070	0.031
	p_adj	0.987	0.987	0.987	0.987	0.987	0.987	0.987	0.987	0.987	0.987	0.987	0.987	0.987	0.987	0.716	0.987	0.987	0.987
Time SL Peak P	R	0.051	0.045	0.077	0.002	0.035	0.070	0.072	-0.016	0.026	-0.034	0.122	0.025	0.033	-0.092	-0.131	-0.044	-0.170	0.017
	p_adj	0.987	0.987	0.987	0.990	0.987	0.987	0.987	0.987	0.987	0.987	0.987	0.987	0.987	0.987	0.987	0.987	0.987	0.987
SC Peak G	R	-0.071	-0.021	-0.160	0.077	-0.101	-0.223	-0.024	-0.125	-0.074	-0.026	-0.073	-0.060	-0.034	0.082	0.038	-0.140	-0.241	-0.174
	p_adj	0.961	0.987	0.961	0.961	0.961	0.821	0.987	0.961	0.961	0.987	0.961	0.961	0.987	0.961	0.987	0.961	0.821	0.961
Time SC Peak G	R	0.106	-0.015	0.082	0.116	0.129	0.065	0.234	0.032	-0.159	0.078	-0.234	-0.185	0.030	-0.168	-0.110	-0.030	0.159	0.066
	p_adj	0.961	0.989	0.961	0.961	0.961	0.961	0.821	0.987	0.961	0.961	0.821	0.961	0.987	0.961	0.961	0.987	0.961	0.961
SC Peak S	R	-0.064	-0.041	-0.283	0.045	-0.001	-0.158	0.029	0.086	-0.082	-0.009	-0.007	0.016	-0.060	-0.075	0.151	-0.144	-0.219	-0.132
	p_adj	0.961	0.987	0.821	0.975	0.997	0.961	0.987	0.961	0.961	0.989	0.989	0.989	0.961	0.961	0.961	0.961	0.821	0.961
Time SC Peak S	R	0.008	-0.128	0.013	0.068	0.053	-0.118	-0.095	-0.021	-0.161	-0.056	-0.119	-0.120	-0.299	-0.092	-0.062	-0.074	-0.118	-0.241
	p_adj	0.989	0.961	0.989	0.961	0.961	0.961	0.961	0.987	0.961	0.961	0.961	0.961	0.821	0.961	0.961	0.961	0.961	0.821
SC Peak P	R	-0.060	-0.014	0.141	0.098	-0.045	-0.242	-0.128	0.055	0.054	0.104	-0.083	-0.128	0.082	0.144	0.045	-0.081	-0.145	0.008
	p_adj	0.961	0.989	0.961	0.961	0.975	0.821	0.961	0.961	0.961	0.961	0.961	0.961	0.961	0.961	0.975	0.961	0.961	0.989
Time SC Peak P	R	-0.154	-0.005	-0.019	0.064	-0.082	-0.013	0.069	0.157	-0.172	-0.222	-0.109	-0.021	0.025	0.127	-0.062	-0.092	0.011	-0.105
	p_adj	0.961	0.989	0.989	0.961	0.961	0.989	0.961	0.961	0.961	0.821	0.961	0.987	0.987	0.961	0.961	0.961	0.989	0.961
SR Peak G	R	-0.146	-0.145	-0.146	-0.059	0.041	-0.018	-0.124	-0.209	-0.272	-0.220	-0.125	-0.080	-0.072	-0.117	-0.126	-0.084	0.001	0.036
	p_adj	0.961	0.961	0.961	0.961	0.987	0.989	0.961	0.838	0.821	0.821	0.961	0.961	0.961	0.961	0.961	0.961	0.997	0.987
Time SR Peak G	R	0.068	0.001	0.017	0.125	0.148	0.075	0.067	0.229	0.211	0.068	0.160	-0.068	-0.072	-0.048	0.097	0.023	-0.033	0.095
	p_adj	0.961	0.997	0.989	0.961	0.961	0.961	0.961	0.821	0.830	0.961	0.961	0.961	0.961	0.975	0.961	0.987	0.987	0.961

R - coefficient of Spearman's rank correlation, p_adj - p-value with FDR (false discovery rate) correction for multiple comparisons

Table S2. Comparison infants with an open (n = 46) and closed (n = 19) arterial duct.

Parameter	P - value*
SL Peak G	0.079
Time SL Peak G	0.099
SL Peak S	0.146
Time SL Peak S	0.203
SL Peak P	0.427
Time SL Peak P	0.578
SC Peak G	0.827
Time SC Peak G	0.859
SC Peak S	0.892
Time SC Peak S	0.089
SC Peak P	0.117
Time SC Peak P	0.181
SR Peak G	0.218
Time SR Peak G	0.448

* Permutation test for the entire set of segments

Table S3. Percentile tables for segmental strain characteristics

Longitudinal strain		Peak Value					Time to Peak				
	Segment	p05	p25	p50	p75	p95	p05	p25	p50	p75	p95
SL Peak S	MV_AntSept	-18.5	-16.4	-12.9	-9.1	-0.5	7.4	164.0	185.0	209.0	274.0
	MV_Ant	-18.1	-13.2	-10.0	-6.7	-0.6	46.0	164.0	193.0	226.3	285.2
	MV_Lat	-18.3	-9.8	-4.8	-1.3	0.0	0.0	177.0	193.0	209.0	285.0
	MV_Post	-18.7	-13.8	-8.3	-3.0	0.0	0.0	163.8	186.0	209.0	281.0
	MV_Inf	-21.3	-14.1	-9.3	-5.6	-0.3	22.0	161.0	185.0	217.0	289.0
	MV_Sept	-21.2	-17.9	-15.6	-11.9	-7.1	0.0	166.0	189.5	211.0	280.8
	PM_AntSept	-23.4	-19.8	-15.5	-11.6	-0.5	7.4	169.0	187.0	211.0	291.8
	PM_Ant	-18.6	-15.9	-11.9	-7.6	-3.6	46.0	159.3	194.0	221.8	274.6
	PM_Lat	-20.4	-14.9	-9.8	-5.2	-0.7	152.4	185.0	201.0	225.0	292.9
	PM_Post	-21.7	-16.5	-12.7	-6.5	-0.1	0.0	176.8	199.5	217.5	281.7
	PM_Inf	-21.6	-19.1	-14.2	-10.1	-5.5	48.0	169.0	193.0	219.0	289.4
	PM_Sept	-24.2	-19.8	-17.6	-13.0	-7.5	128.8	185.0	198.0	225.0	284.7
	AP_AntSept	-33.7	-24.3	-20.3	-12.9	-0.4	7.4	176.8	196.0	217.0	294.4
	AP_Ant	-26.9	-20.8	-17.1	-11.3	-5.7	46.4	164.0	194.0	227.0	284.1
	AP_Lat	-27.0	-21.8	-18.0	-12.5	-4.5	152.0	191.5	203.0	225.0	296.3
	AP_Post	-30.4	-23.9	-18.3	-10.7	-0.5	7.4	176.0	197.5	217.0	294.1
	AP_Inf	-26.5	-23.7	-18.9	-13.2	-4.3	47.6	168.3	193.0	227.0	293.6
	AP_Sept	-29.9	-24.7	-20.4	-15.3	-6.7	154.8	185.0	201.0	225.0	296.3

Longitudinal strain		Peak Value					Time to Peak				
	Segment	p05	p25	p50	p75	p95	p05	p25	p50	p75	p95
SL Peak P	MV_AntSept	0.0	0.0	0.4	3.2	7.2	0.0	0.0	9.0	49.8	98.7
	MV_Ant	0.0	0.0	0.1	4.0	7.4	0.0	0.0	35.5	56.0	117.2
	MV_Lat	0.0	0.9	2.4	4.6	11.5	0.0	8.0	33.0	75.5	137.0
	MV_Post	0.0	0.2	1.7	4.7	12.1	0.0	0.0	32.0	57.3	121.0
	MV_Inf	0.0	0.0	0.5	3.3	12.5	0.0	0.0	22.0	64.0	145.0
	MV_Sept	0.0	0.0	0.4	2.4	9.4	0.0	8.0	39.0	68.0	119.0
	PM_AntSept	0.0	0.0	0.2	5.4	11.2	0.0	0.0	0.0	49.8	97.5
	PM_Ant	0.0	0.0	0.1	2.8	6.7	0.0	0.0	32.0	55.0	82.3
	PM_Lat	0.0	0.3	2.0	3.8	10.9	0.0	0.0	24.0	56.8	144.0
	PM_Post	0.0	0.0	1.1	3.1	12.2	0.0	0.0	31.0	55.0	126.6
	PM_Inf	0.0	0.0	0.1	1.2	12.9	0.0	0.0	8.0	48.0	97.1
	PM_Sept	0.0	0.0	0.4	3.1	11.7	0.0	0.0	32.0	64.0	118.2
	AP_AntSept	0.0	0.0	0.4	9.2	19.4	0.0	0.0	11.0	55.0	117.7
	AP_Ant	0.0	0.0	0.0	0.6	11.0	0.0	0.0	9.0	46.8	88.1
	AP_Lat	0.0	0.3	1.6	8.2	25.3	0.0	0.0	28.0	55.3	145.0
	AP_Post	0.0	0.0	0.3	8.8	20.0	0.0	0.0	18.5	55.3	119.4
	AP_Inf	0.0	0.0	0.0	2.1	17.8	0.0	0.0	0.0	49.8	98.1
	AP_Sept	0.0	0.1	1.1	8.2	25.4	0.0	9.5	32.0	58.0	137.0

Longitudinal strain		Peak Value					Time to Peak				
	Segment	p05	p25	p50	p75	p95	p05	p25	p50	p75	p95
SL Peak G	MV_AntSept	-18.6	-16.9	-13.1	-10.0	-6.3	143.0	176.0	193.0	233.0	306.7
	MV_Ant	-18.7	-13.8	-10.5	-7.7	-3.3	151.4	193.0	231.5	258.5	296.5
	MV_Lat	-20.2	-12.0	-6.5	-2.7	-0.3	152.0	195.0	230.0	264.5	375.8
	MV_Post	-20.6	-15.9	-8.9	-4.5	-0.5	95.3	177.0	209.0	252.5	352.0
	MV_Inf	-21.7	-15.9	-10.0	-7.2	-1.8	143.0	176.0	209.0	263.0	329.0
	MV_Sept	-21.6	-18.2	-15.9	-12.4	-7.8	155.8	177.0	209.0	264.0	337.0
	PM_AntSept	-23.6	-20.4	-15.9	-12.0	-6.1	137.9	183.0	194.0	235.0	317.9
	PM_Ant	-20.0	-16.1	-12.3	-8.4	-5.8	152.6	192.8	227.5	258.8	298.6
	PM_Lat	-21.4	-15.8	-12.2	-6.7	-2.3	177.0	213.3	241.0	267.0	347.8
	PM_Post	-22.0	-18.1	-14.2	-8.3	-1.8	153.4	195.0	233.0	257.0	359.5
	PM_Inf	-22.2	-19.2	-15.1	-10.8	-6.5	152.6	176.8	201.0	235.0	305.4
	PM_Sept	-24.3	-19.9	-18.0	-14.5	-9.8	169.0	191.5	219.0	250.3	325.6
	AP_AntSept	-33.9	-25.7	-21.1	-15.4	-7.4	153.2	185.0	218.0	249.0	331.6
	AP_Ant	-27.3	-21.8	-17.3	-11.6	-8.6	143.0	169.0	222.0	257.0	313.3
	AP_Lat	-28.8	-24.8	-19.9	-14.0	-7.3	167.1	217.0	241.0	269.0	341.3
	AP_Post	-31.2	-25.4	-20.3	-13.7	-4.6	152.3	197.3	233.0	267.0	366.6
	AP_Inf	-27.0	-23.7	-18.9	-13.6	-8.4	151.6	176.8	201.0	241.0	313.3
	AP_Sept	-30.8	-26.2	-21.8	-16.0	-9.3	171.5	208.8	242.5	265.0	347.9

Circumferentinal strain		Peak Value					Time to Peak				
	Segment	p05	p25	p50	p75	p95	p05	p25	p50	p75	p95
SC Peak S	MV_AntSept	-28.4	-22.2	-17.6	-12.2	-3.1	0.0	72.0	169.0	198.0	265.0
	MV_Ant	-20.8	-12.5	-6.7	-0.5	0.0	0.0	33.0	121.0	185.0	226.8
	MV_Lat	-21.1	-8.6	-4.7	-0.6	0.0	0.0	81.0	129.0	177.0	215.4
	MV_Post	-13.7	-7.7	-3.8	-0.9	0.0	8.0	143.0	169.0	187.0	254.6
	MV_Inf	-17.5	-9.8	-6.8	-4.2	-0.3	6.4	143.0	177.0	201.0	265.2
	MV_Sept	-23.7	-20.0	-16.2	-11.5	-3.2	0.0	64.0	176.0	201.0	252.2
	PM_AntSept	-22.8	-15.1	-12.1	-8.6	-0.1	0.0	44.0	157.0	196.5	265.0
	PM_Ant	-17.1	-7.3	-3.4	-0.6	0.0	0.0	48.0	169.0	205.0	255.0
	PM_Lat	-16.9	-7.5	-3.9	-1.4	0.0	30.0	123.5	163.0	195.8	245.3
	PM_Post	-14.2	-7.3	-4.7	-1.3	0.0	52.5	144.5	177.0	193.0	267.3
	PM_Inf	-15.2	-10.2	-7.7	-5.0	-1.4	0.0	125.5	177.0	198.8	263.3
	PM_Sept	-22.3	-18.4	-15.8	-12.6	-2.6	0.0	111.5	177.0	196.5	267.4
	AP_AntSept	-23.3	-15.2	-11.9	-5.8	0.0	0.0	20.5	144.0	194.3	257.5
	AP_Ant	-19.2	-9.6	-5.0	-0.6	0.0	0.0	36.0	167.5	198.8	255.3
	AP_Lat	-14.0	-8.5	-4.4	-0.5	0.0	12.0	152.8	176.5	209.0	273.0
	AP_Post	-18.4	-11.1	-5.4	-0.6	0.0	12.0	154.0	186.0	211.0	266.5
	AP_Inf	-25.5	-16.9	-10.9	-7.6	-1.6	52.2	169.0	185.0	212.0	286.2
	AP_Sept	-23.0	-19.4	-15.3	-10.6	-4.6	0.0	54.0	177.0	207.0	255.9
**Circumferentinal strain**		Peak Value					Time to Peak				
	Segment	p05	p25	p50	p75	p95	p05	p25	p50	p75	p95
SC Peak P	MV_AntSept	0.0	0.0	0.1	5.2	12.0	0.0	8.0	40.0	72.0	160.0
	MV_Ant	0.0	1.1	3.6	6.7	13.6	0.0	11.0	64.0	129.0	234.6
	MV_Lat	0.0	0.4	2.8	6.4	13.9	0.0	0.0	48.0	185.0	225.0
	MV_Post	0.0	0.5	2.5	7.6	20.0	0.0	0.0	0.0	55.0	125.8
	MV_Inf	0.0	0.2	2.1	4.5	15.4	0.0	0.0	0.0	48.0	93.8
	MV_Sept	0.0	0.0	0.0	1.9	8.6	0.0	32.0	49.0	78.0	155.4
	PM_AntSept	0.0	0.0	0.1	4.0	10.0	0.0	28.5	54.5	97.5	173.3
	PM_Ant	0.0	1.3	4.1	8.9	15.6	0.0	22.0	56.0	105.0	199.0
	PM_Lat	0.0	0.8	2.6	6.6	13.5	0.0	0.0	16.0	49.8	129.8
	PM_Post	0.0	0.4	3.4	6.9	13.4	0.0	0.0	0.0	40.0	88.0
	PM_Inf	0.0	0.0	0.5	2.5	5.2	0.0	0.0	32.0	56.8	150.0
	PM_Sept	-0.1	0.0	0.0	0.6	6.2	0.0	0.0	40.0	88.0	166.9
	AP_AntSept	0.0	0.1	1.6	6.0	17.5	0.0	16.0	52.0	102.5	185.3
	AP_Ant	0.0	0.8	5.1	8.0	17.1	0.0	9.5	56.0	90.3	196.0
	AP_Lat	0.0	0.2	2.5	8.4	21.8	0.0	0.0	16.0	64.0	121.0
	AP_Post	0.0	0.1	2.3	6.2	15.3	0.0	0.0	12.0	64.0	93.0
	AP_Inf	0.0	0.0	0.7	4.4	10.4	0.0	0.0	0.0	48.0	69.9
	AP_Sept	0.0	0.0	0.0	4.0	11.7	0.0	11.0	48.0	77.8	131.8
**Circumferentinal strain**		Peak Value					Time to Peak				
	Segment	p05	p25	p50	p75	p95	p05	p25	p50	p75	p95
SC Peak G	MV_AntSept	-29.0	-23.8	-18.5	-13.6	-6.1	79.4	185.0	208.0	283.0	352.2
	MV_Ant	-20.8	-12.7	-8.0	-4.1	0.3	38.6	108.0	185.0	249.0	362.6
	MV_Lat	-21.9	-9.6	-5.0	-2.5	1.8	56.0	110.0	165.0	209.0	327.4
	MV_Post	-14.1	-8.6	-4.6	-2.3	6.9	112.4	152.0	177.0	225.0	303.4
	MV_Inf	-18.1	-10.1	-7.5	-4.5	-1.4	121.0	166.0	195.0	241.0	331.2
	MV_Sept	-23.7	-20.0	-16.2	-12.6	-8.6	123.4	177.0	209.0	273.0	341.0
	PM_AntSept	-22.8	-17.6	-13.2	-9.4	-2.1	89.5	167.0	202.0	263.0	329.0
	PM_Ant	-18.5	-8.8	-5.9	-3.2	-0.9	81.8	167.0	213.0	265.5	337.0
	PM_Lat	-16.9	-9.5	-4.6	-2.5	-0.3	107.0	150.5	186.5	233.0	313.0
	PM_Post	-15.1	-7.7	-5.6	-2.3	-0.8	132.0	164.8	189.5	235.8	289.0
	PM_Inf	-15.2	-10.9	-8.3	-6.1	-3.7	115.5	165.0	193.0	241.0	293.0
	PM_Sept	-22.3	-18.4	-16.5	-12.8	-7.3	103.4	176.0	198.0	258.5	329.0
	AP_AntSept	-23.3	-15.5	-12.6	-7.7	-2.3	19.3	169.0	205.0	273.0	339.0
	AP_Ant	-20.0	-10.9	-7.5	-3.4	3.4	36.0	164.0	208.5	257.0	313.0
	AP_Lat	-14.5	-9.7	-5.7	-3.3	0.0	125.0	164.0	195.5	241.0	281.0
	AP_Post	-18.4	-11.1	-6.2	-2.5	1.6	129.3	164.8	193.0	241.0	289.0
	AP_Inf	-25.8	-16.9	-11.5	-8.2	-5.0	138.5	172.5	193.0	249.0	304.2
	AP_Sept	-23.0	-19.4	-16.0	-11.0	-6.9	122.0	185.0	231.0	279.3	358.6

Radial strain		Peak Value					Time to Peak				
	Segment	p05	p25	p50	p75	p95	p05	p25	p50	p75	p95
SR Peak G	MV_AntSept	3.0	12.4	23.9	39.0	63.5	30.4	193.0	230.0	254.0	342.4
	MV_Ant	2.0	8.0	15.5	35.6	71.6	22.0	208.0	233.0	263.0	343.8
	MV_Lat	0.9	5.3	14.6	25.1	67.3	128.8	201.0	233.0	273.0	321.8
	MV_Post	1.4	10.1	18.6	32.9	69.5	156.4	186.0	217.0	252.0	321.8
	MV_Inf	7.8	14.0	27.8	47.5	83.1	118.4	177.0	209.0	241.0	316.2
	MV_Sept	8.5	19.6	31.7	46.7	62.9	21.4	176.0	219.0	252.0	305.4
	PM_AntSept	2.8	10.3	19.4	29.0	45.5	15.8	177.0	212.5	252.5	319.0
	PM_Ant	0.2	4.6	13.4	30.3	49.4	15.8	176.8	217.0	252.0	318.0
	PM_Lat	0.4	4.7	11.5	25.7	49.9	118.3	176.0	212.0	243.5	300.3
	PM_Post	1.5	9.4	17.7	30.5	58.9	134.3	177.0	205.0	241.0	277.8
	PM_Inf	4.9	16.7	26.7	39.7	61.8	118.8	176.8	217.0	252.0	303.8
	PM_Sept	8.9	20.5	28.6	36.0	46.3	81.4	165.0	201.0	242.5	332.3
	AP_AntSept	3.1	14.3	24.0	47.8	86.0	88.8	161.0	189.0	252.5	299.0
	AP_Ant	1.9	9.4	22.7	42.3	82.8	68.8	161.0	193.0	257.0	303.5
	AP_Lat	2.5	7.7	16.8	41.7	76.5	20.8	164.8	187.0	233.5	291.0
	AP_Post	0.2	9.3	17.7	39.4	75.4	32.8	168.3	193.0	235.0	281.0
	AP_Inf	1.7	14.8	19.3	40.8	87.2	116.6	176.5	201.0	241.0	296.3
	AP_Sept	4.1	16.0	28.3	40.3	78.1	77.1	185.3	233.0	257.0	372.0

p05 5th percentile
p25 25th percentile (Q1)
p50 50th percentile (median)
p75 75th percentile (Q3)
p95 95th percentile

Table S4. Correlation between Peaks and Time to Peaks

Longitudinal strain

Segment	SL Peak S vs Time to SL Peak S		SL Peak G vs Time to SL Peak G		SL Peak P vs Time to SL Peak P		SL Peak S vs SL Peak G		Time to SL Peak S vs Time to SL Peak G	
	R	p_adj	R	p_adj	R	p_adj	R	p_adj	R	p_adj
MV_AntSept	-0.172	0.218	0.120	0.344	***0.710***	***0.000***	***0.906***	***0.000***	***0.677***	***0.000***
MV_Ant	0.115	0.380	***0.372***	***0.004***	***0.585***	***0.000***	***0.895***	***0.000***	***0.547***	***0.000***
MV_Lat	***-0.358***	***0.004***	-0.110	0.377	***0.409***	***0.001***	***0.929***	***0.000***	***0.374***	***0.003***
MV_Post	-0.308	0.017	-0.054	0.670	***0.626***	***0.000***	***0.948***	***0.000***	***0.588***	***0.000***
MV_Inf	-0.041	0.755	0.213	0.125	***0.680***	***0.000***	***0.946***	***0.000***	***0.588***	***0.000***
MV_Sept	0.096	0.436	***0.279***	***0.026***	***0.479***	***0.000***	***0.946***	***0.000***	***0.371***	***0.003***
PM_AntSept	-0.197	0.148	0.100	0.433	***0.799***	***0.000***	***0.927***	***0.000***	***0.713***	***0.000***
PM_Ant	0.107	0.418	***0.316***	***0.017***	***0.515***	***0.000***	***0.900***	***0.000***	***0.478***	***0.000***
PM_Lat	0.132	0.284	***0.306***	***0.014***	***0.687***	***0.000***	***0.959***	***0.000***	***0.569***	***0.000***
PM_Post	-0.185	0.179	0.086	0.500	***0.634***	***0.000***	***0.952***	***0.000***	***0.688***	***0.000***
PM_Inf	0.212	0.103	***0.422***	***0.001***	***0.550***	***0.000***	***0.923***	***0.000***	***0.743***	***0.000***
PM_Sept	0.123	0.319	0.243	0.057	***0.556***	***0.000***	***0.949***	***0.000***	***0.730***	***0.000***
AP_AntSept	***-0.268***	***0.040***	-0.003	0.981	***0.741***	***0.000***	***0.928***	***0.000***	***0.725***	***0.000***
AP_Ant	0.100	0.447	0.235	0.088	***0.507***	***0.000***	***0.905***	***0.000***	***0.591***	***0.000***
AP_Lat	0.113	0.359	***0.253***	***0.047***	***0.775***	***0.000***	***0.931***	***0.000***	***0.678***	***0.000***
AP_Post	-0.208	0.123	0.100	0.433	***0.657***	***0.000***	***0.926***	***0.000***	***0.618***	***0.000***
AP_Inf	0.090	0.492	0.220	0.115	***0.553***	***0.000***	***0.915***	***0.000***	***0.764***	***0.000***
AP_Sept	0.132	0.281	0.205	0.116	***0.682***	***0.000***	***0.948***	***0.000***	***0.562***	***0.000***

Circumferentinal strain

Segment	SC Peak S vs Time to SC Peak S		SC Peak G vs Time to SC Peak G		SC Peak P vs Time to SC Peak P		SC Peak S vs SC Peak G		Time to SC Peak S vs Time to SC Peak G	
	R	p_adj	R	p_adj	R	p_adj	R	p_adj	R	p_adj
MV_AntSept	-0.042	0.756	0.078	0.674	0.314	0.053	***0.928***	***0.000***	0.186	0.332
MV_Ant	0.102	0.541	0.146	0.417	0.219	0.204	***0.927***	***0.000***	0.222	0.204
MV_Lat	-0.077	0.592	0.218	0.181	0.208	0.181	***0.894***	***0.000***	0.349	0.023
MV_Post	-0.151	0.527	-0.103	0.640	0.046	0.732	***0.894***	***0.000***	***0.562***	***0.000***
MV_Inf	-0.065	0.759	-0.126	0.527	0.018	0.894	***0.887***	***0.000***	***0.475***	***0.001***
MV_Sept	0.021	0.882	-0.034	0.882	***0.398***	***0.007***	***0.989***	***0.000***	0.080	0.882
PM_AntSept	-0.175	0.296	0.133	0.393	***0.444***	***0.002***	***0.876***	***0.000***	0.205	0.261
PM_Ant	-0.132	0.332	0.168	0.332	0.139	0.332	***0.754***	***0.000***	0.284	0.102
PM_Lat	-0.145	0.343	-0.115	0.398	0.194	0.302	***0.941***	***0.000***	***0.552***	***0.000***
PM_Post	-0.020	0.882	0.129	0.517	0.322	0.031	***0.927***	***0.000***	***0.591***	***0.000***
PM_Inf	-0.074	0.707	0.219	0.156	0.287	0.064	***0.845***	***0.000***	0.307	0.064
PM_Sept	-0.159	0.370	-0.139	0.375	0.289	0.064	***0.932***	***0.000***	***0.328***	***0.043***
AP_AntSept	-0.277	0.078	0.091	0.506	0.310	0.061	***0.913***	***0.000***	0.229	0.135
AP_Ant	-0.180	0.278	-0.049	0.719	0.296	0.054	***0.884***	***0.000***	***0.415***	***0.004***
AP_Lat	-0.233	0.125	-0.166	0.264	0.301	0.048	***0.820***	***0.000***	***0.526***	***0.000***
AP_Post	-0.093	0.498	0.139	0.462	***0.493***	***0.000***	***0.929***	***0.000***	***0.622***	***0.000***
AP_Inf	-0.019	0.891	0.057	0.813	0.080	0.813	***0.963***	***0.000***	***0.728***	***0.000***
AP_Sept	0.069	0.804	-0.056	0.804	0.232	0.274	***0.946***	***0.000***	-0.035	0.804

Radial strain

Segment	SR Peak G vs Time to SR Peak G	
	R	p_adj
MV_AntSept	-0.082	0.674
MV_Ant	0.042	0.758
MV_Lat	0.072	0.592
MV_Post	-0.084	0.640
MV_Inf	-0.145	0.527

MV_Sept	-0.020	0.882
PM_AntSept	0.018	0.895
PM_Ant	0.148	0.332
PM_Lat	-0.146	0.343
PM_Post	-0.102	0.547
PM_Inf	-0.037	0.788
PM_Sept	-0.059	0.666
AP_AntSept	-0.191	0.190
AP_Ant	-0.077	0.686
AP_Lat	-0.122	0.369
AP_Post	-0.099	0.498
AP_Inf	-0.184	0.357
AP_Sept	0.046	0.804

R - Spearman's rank coefficient correlation of Spearmen, p_adj - FDR-corrected p-value

Table S5. Correlation between: Peak P and Peaks S , G; Time to Peak P and Time to Peaks S, G

Longitudinal strain

Segment	SLPeakS vs SLPeakP		Time to SLPeakS vs Time to SLPeakP		SLPeakG vs SLPeakP		Time to SLPeakG vs Time to SLPeakP	
	R	p_adj	R	p_adj	R	p_adj	R	p_adj
MV_AntSept	***0.520***	***0.000***	***0.370***	***0.003***	***0.600***	***0.000***	***0.409***	***0.001***
MV_Ant	***0.467***	***0.000***	***0.403***	***0.002***	***0.375***	***0.003***	***0.516***	***0.000***
MV_Lat	***0.617***	***0.000***	0.083	0.589	***0.511***	***0.000***	***0.238***	***0.074***
MV_Post	***0.539***	***0.000***	0.221	0.093	***0.555***	***0.000***	***0.279***	***0.036***
MV_Inf	***0.471***	***0.000***	***0.284***	***0.026***	***0.454***	***0.000***	***0.395***	***0.002***
MV_Sept	***0.580***	***0.000***	0.093	0.451	***0.514***	***0.000***	***0.250***	***0.047***
PM_AntSept	***0.553***	***0.000***	***0.417***	***0.001***	***0.570***	***0.000***	***0.504***	***0.000***
PM_Ant	***0.465***	***0.000***	***0.438***	***0.001***	***0.416***	***0.001***	***0.400***	***0.002***
PM_Lat	***0.666***	***0.000***	***0.466***	***0.000***	***0.631***	***0.000***	***0.528***	***0.000***
PM_Post	***0.576***	***0.000***	0.262	0.051	***0.607***	***0.000***	0.217	0.099
PM_Inf	***0.575***	***0.000***	***0.388***	***0.003***	***0.574***	***0.000***	***0.443***	***0.001***
PM_Sept	***0.551***	***0.000***	***0.319***	***0.008***	***0.490***	***0.000***	***0.401***	***0.001***
AP_AntSept	***0.425***	***0.001***	***0.428***	***0.001***	***0.395***	***0.001***	***0.563***	***0.000***
AP_Ant	0.203	0.116	***0.427***	***0.001***	0.252	0.059	***0.435***	***0.001***
AP_Lat	***0.576***	***0.000***	***0.425***	***0.000***	***0.480***	***0.000***	***0.527***	***0.000***
AP_Post	***0.440***	***0.001***	***0.402***	***0.001***	***0.360***	***0.004***	***0.438***	***0.001***
AP_Inf	***0.321***	***0.016***	***0.519***	***0.000***	***0.309***	***0.018***	***0.365***	***0.007***
AP_Sept	***0.517***	***0.000***	***0.310***	***0.010***	***0.426***	***0.000***	***0.618***	***0.000***

Circumferentinal strain

Segment	SC PeakS vs SC PeakP		Time to SC PeakS vs Time to SC PeakP		SC PeakG vs SC PeakP		Time to SC PeakG vs Time to SC PeakP	
	R	p_adj	R	p_adj	R	p_adj	R	p_adj
MV_AntSept	***0.391***	***0.005***	-0.274	0.054	***0.398***	***0.005***	***0.391***	***0.005***
MV_Ant	***0.665***	***0.000***	-0.292	0.053	***0.646***	***0.000***	***0.665***	***0.000***
MV_Lat	***0.685***	***0.000***	-0.296	0.054	***0.672***	***0.000***	***0.685***	***0.000***
MV_Post	***0.704***	***0.000***	0.058	0.666	***0.714***	***0.000***	***0.704***	***0.000***
MV_Inf	***0.525***	***0.000***	***0.316***	***0.017***	***0.558***	***0.000***	***0.525***	***0.000***
MV_Sept	0.224	0.131	-0.091	0.502	0.221	0.131	0.224	0.131
PM_AntSept	***0.408***	***0.004***	-0.201	0.138	***0.434***	***0.003***	***0.408***	***0.004***
PM_Ant	***0.594***	***0.000***	-0.029	0.834	***0.434***	***0.002***	***0.594***	***0.000***
PM_Lat	***0.528***	***0.000***	0.257	0.056	***0.479***	***0.000***	***0.528***	***0.000***
PM_Post	***0.582***	***0.000***	***0.439***	***0.001***	***0.543***	***0.000***	***0.582***	***0.000***
PM_Inf	***0.382***	***0.005***	-0.062	0.652	***0.447***	***0.001***	***0.382***	***0.005***
PM_Sept	0.121	0.380	-0.129	0.380	0.197	0.300	0.121	0.380
AP_AntSept	***0.497***	***0.000***	***0.342***	***0.013***	***0.506***	***0.000***	***0.497***	***0.000***
AP_Ant	***0.681***	***0.000***	-0.239	0.102	***0.604***	***0.000***	***0.681***	***0.000***
AP_Lat	***0.693***	***0.000***	0.129	0.342	***0.583***	***0.000***	***0.693***	***0.000***
AP_Post	***0.642***	***0.000***	***0.379***	***0.004***	***0.564***	***0.000***	***0.642***	***0.000***
AP_Inf	***0.433***	***0.001***	***0.440***	***0.001***	***0.432***	***0.001***	***0.433***	***0.001***
AP_Sept	***0.350***	***0.013***	-0.154	0.268	***0.421***	***0.003***	***0.350***	***0.013***

Table S6. Comparison Longitudinal and Circumferential strain

SL_PeakG vs SC_PeakG		
Segment	Difference Median [Q1;Q3]	p_adj
MV_AntSept	4.67 [0.08; 9.87]	0.000 ***
MV_Ant	-2.66 [-6.37; 1.35]	0.008 **
MV_Lat	-0.03 [-6.82; 5.21]	0.735 ns
MV_Post	-4.89 [-10.72; 0.70]	0.000 ***
MV_Inf	-2.38 [-8.25; 2.31]	0.066 ns
MV_Sept	0.65 [-1.77; 3.44]	0.248 ns
PM_AntSept	-3.28 [-7.19; 1.36]	0.002 **
PM_Ant	-6.66 [-9.52; -2.65]	0.000 ***
PM_Lat	-3.14 [-9.87; 0.25]	0.000 ***
PM_Post	-8.11 [-12.47; -1.42]	0.000 ***
PM_Inf	-6.02 [-9.36; -3.13]	0.000 ***
PM_Sept	-1.07 [-5.50; 2.65]	0.131 ns
AP_AntSept	-8.95 [-16.01; -2.96]	0.000 ***
AP_Ant	-9.22 [-14.98; -3.86]	0.000 ***
AP_Lat	-12.51 [-18.02; -5.75]	0.000 ***
AP_Post	-12.02 [-19.60; -5.29]	0.000 ***
AP_Inf	-4.77 [-11.21; 0.18]	0.000 ***
AP_Sept	-5.74 [-10.26; -1.66]	0.000 ***
**TimeSL_PeakG vs TimeSC_PeakG**		
Segment	Difference Median [Q1;Q3]	p_adj
MV_AntSept	-11.00 [-78.00; 56.00]	0.234 ns
MV_Ant	59.00 [-24.00; 112.00]	0.002 **
MV_Lat	72.00 [10.00; 120.00]	0.000 ***
MV_Post	31.00 [-8.00; 87.00]	0.003 **
MV_Inf	12.00 [-24.00; 55.75]	0.138 ns
MV_Sept	0.00 [-48.00; 22.00]	0.362 ns
PM_AntSept	-8.00 [-45.00; 41.00]	0.620 ns
PM_Ant	-13.00 [-40.75; 71.50]	0.441 ns
PM_Lat	44.50 [6.00; 80.00]	0.000 ***
PM_Post	40.00 [6.00; 65.75]	0.000 ***
PM_Inf	13.50 [-26.00; 44.25]	0.201 ns
PM_Sept	0.00 [-40.50; 65.00]	0.201 ns
AP_AntSept	12.50 [-49.75; 76.00]	0.234 ns
AP_Ant	6.50 [-40.00; 57.75]	0.245 ns
AP_Lat	40.00 [9.50; 82.00]	0.000 ***
AP_Post	32.00 [8.00; 64.00]	0.000 ***
AP_Inf	9.50 [-16.00; 41.00]	0.422 ns
AP_Sept	14.00 [-44.00; 64.00]	0.252 ns

p_adj - FDR-corrected p-value

Figure S1. Gradient of median values of longitudinal strain peaks from base to apex

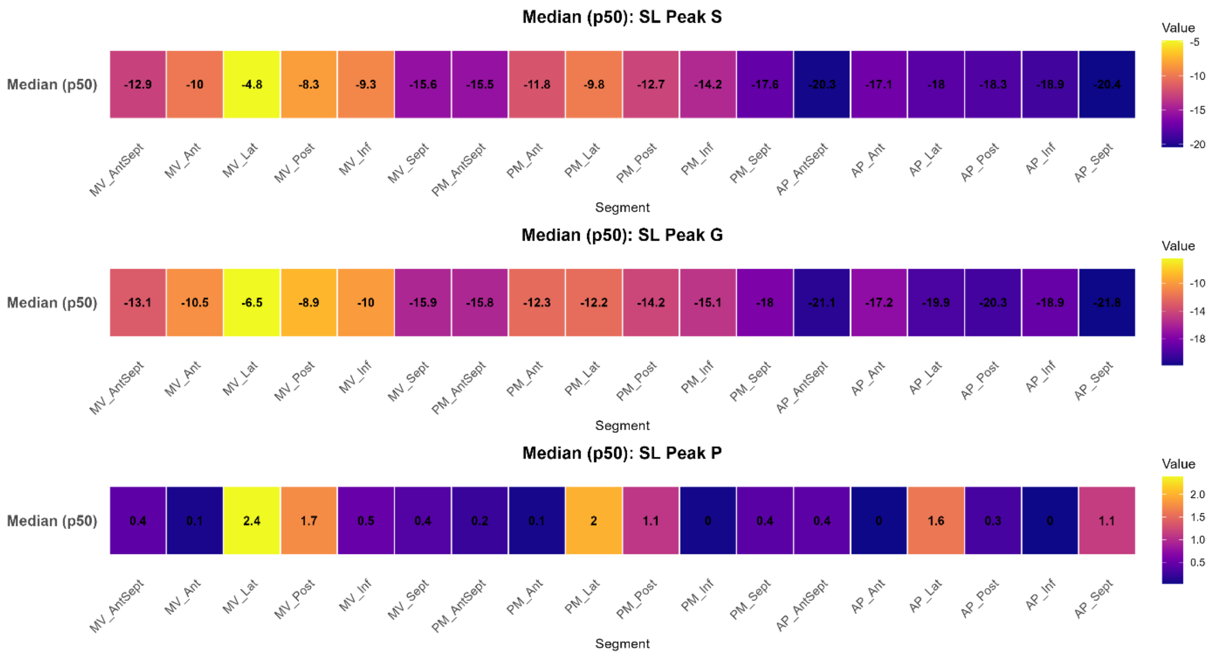

Figure S2. The magnitude and significance of differences in regional characteristics of the strain with the global median

A. Longitudinal strain

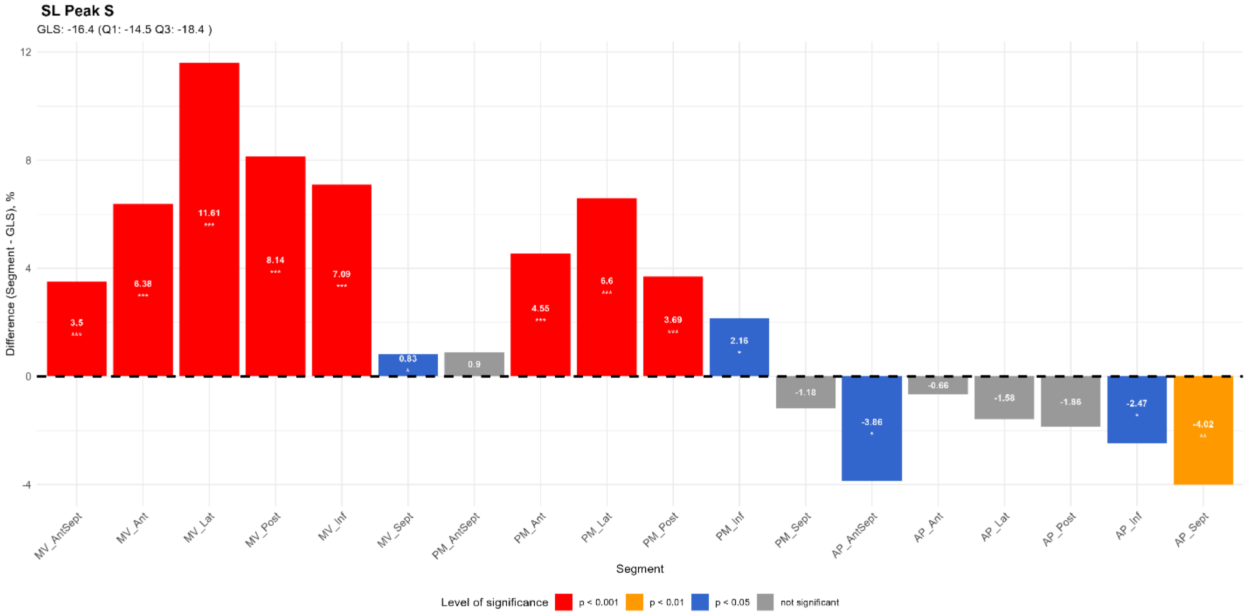

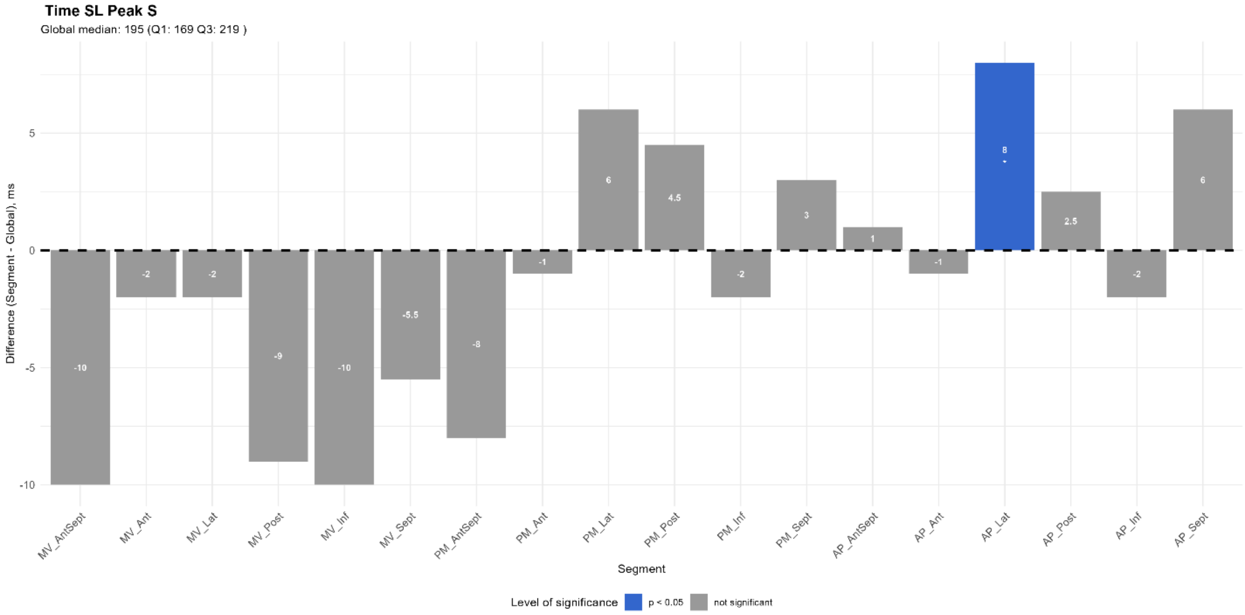

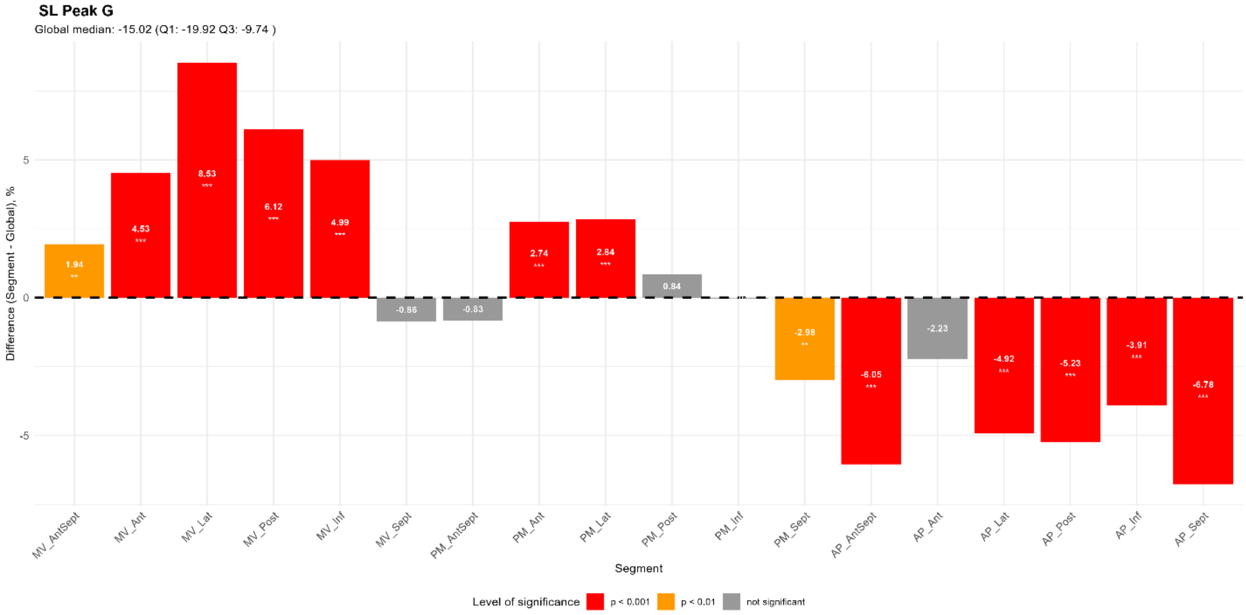

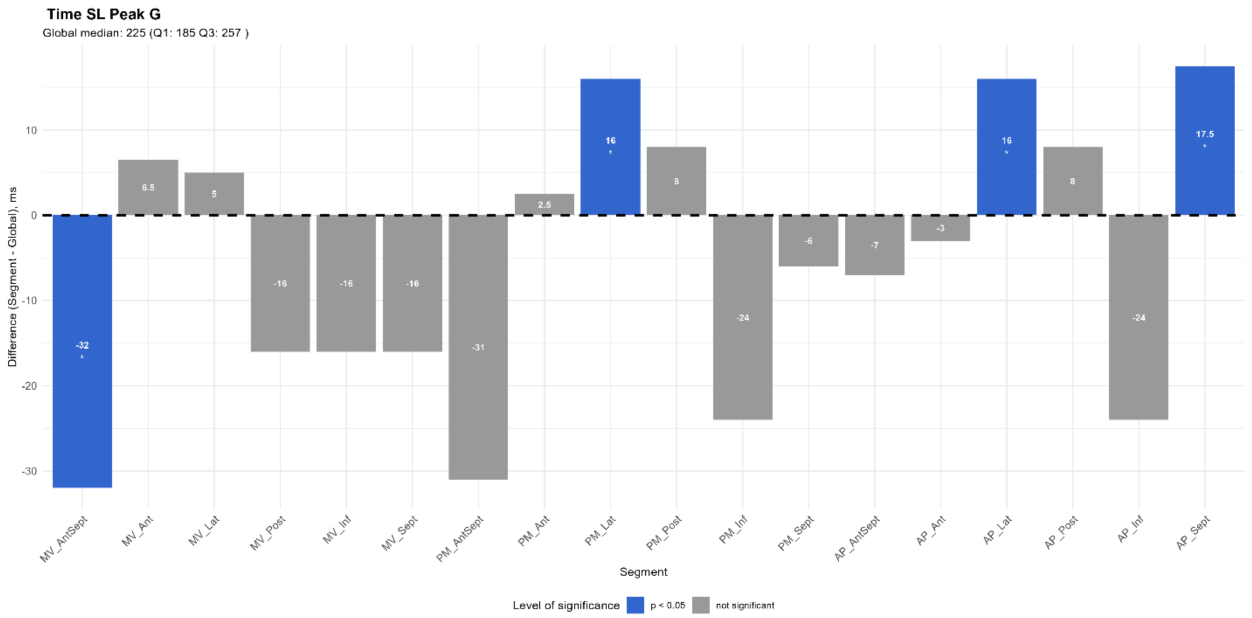
Time SL Peak G
Global median: 225 (Q1: 185 Q3: 257)
Difference (Segment - Global), ms
Segment
Level of significance
p < 0.05
not significant

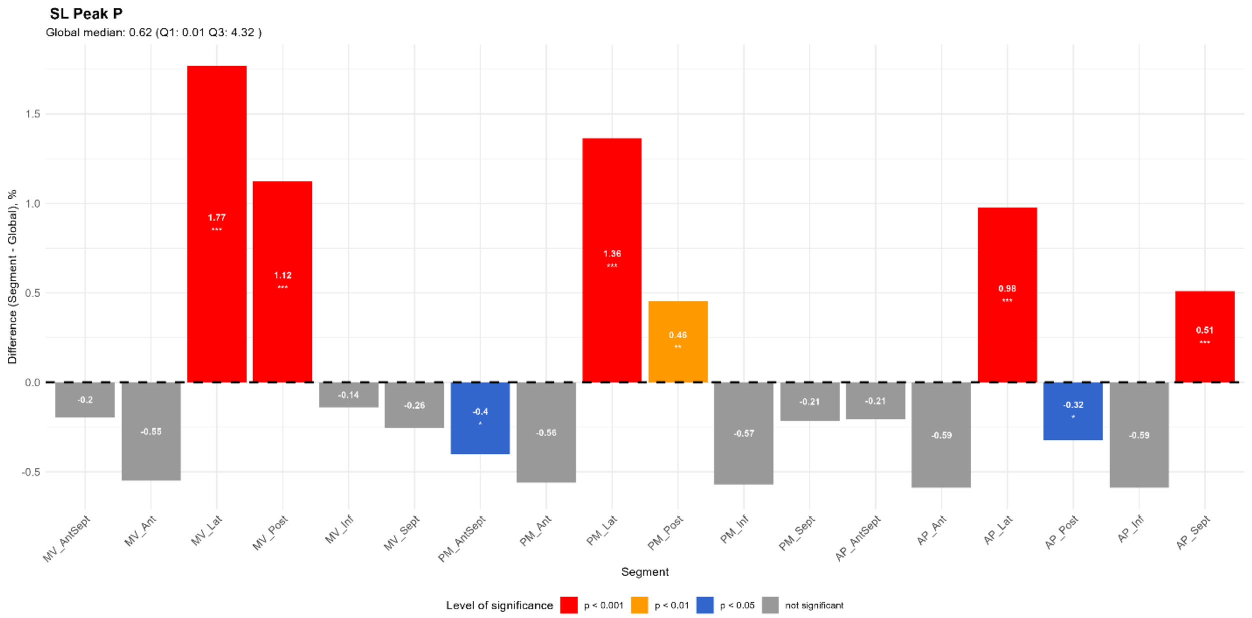
SL Peak P
Global median: 0.62 (Q1: 0.01 Q3: 4.32)
Difference (Segment - Global), %
Segment
Level of significance
p < 0.001
p < 0.01
p < 0.05
not significant

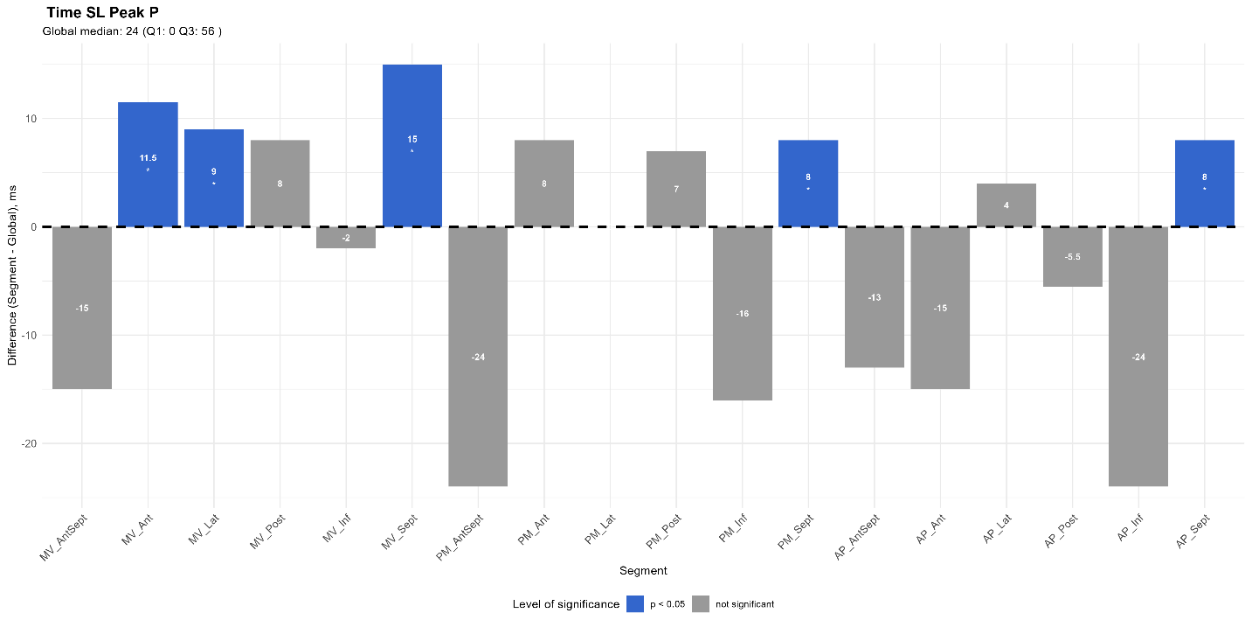
Time SL Peak P
Global median: 24 (Q1: 0 Q3: 56)
Difference (Segment - Global), ms
Segment
Level of significance
p < 0.05
not significant

B. Circumferential strain

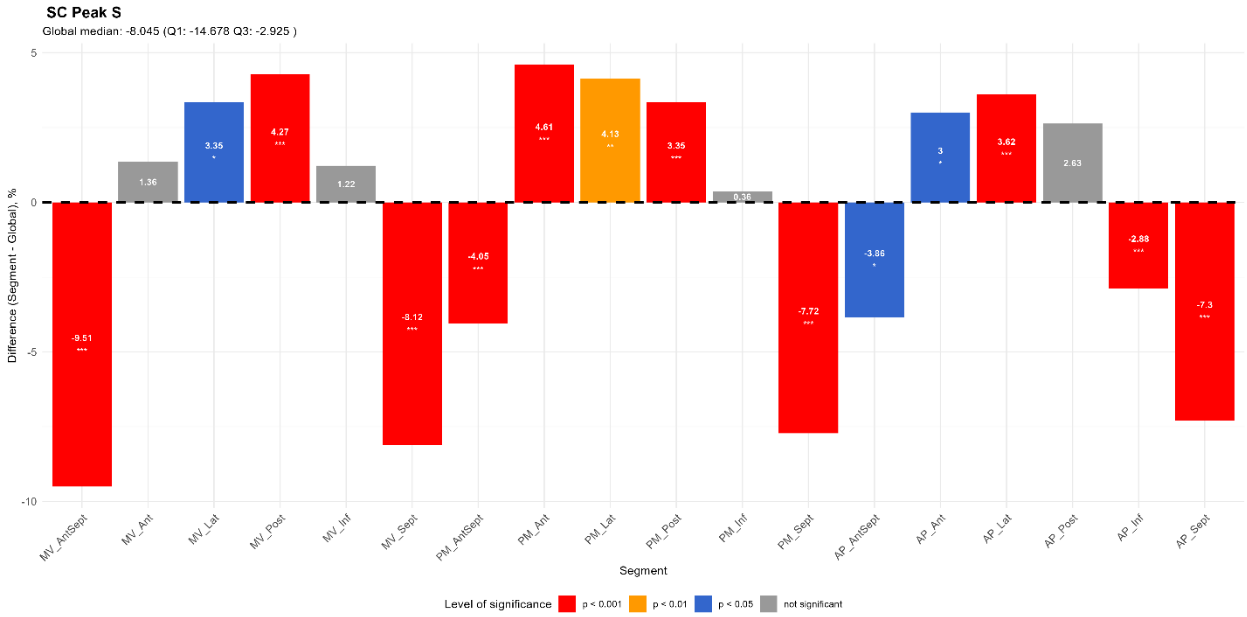

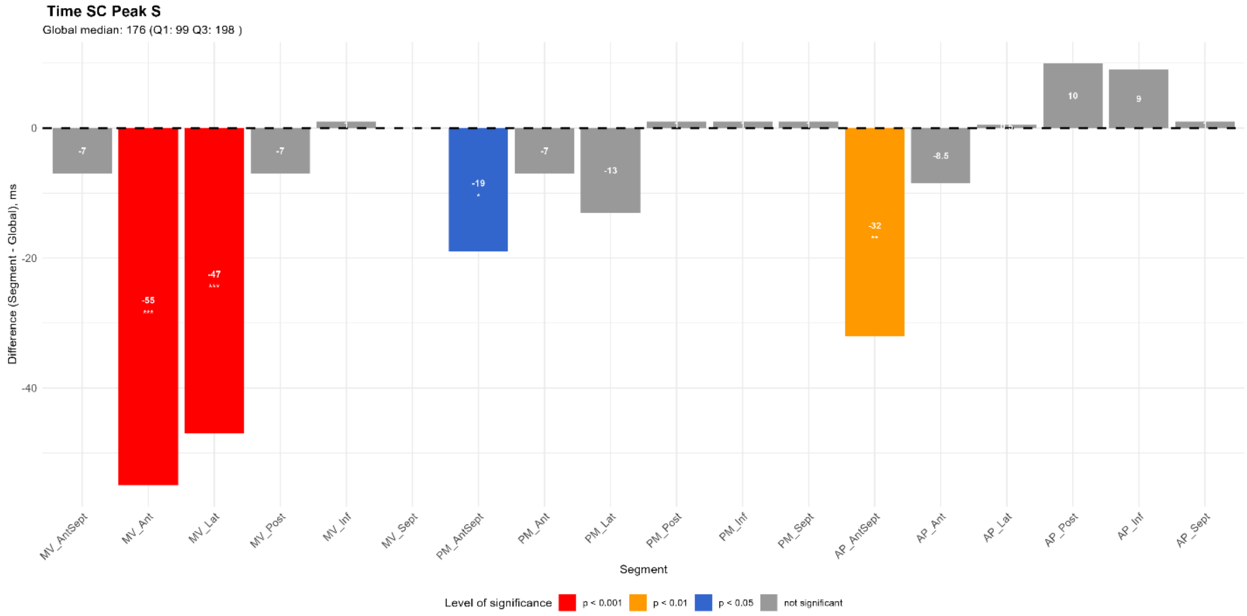

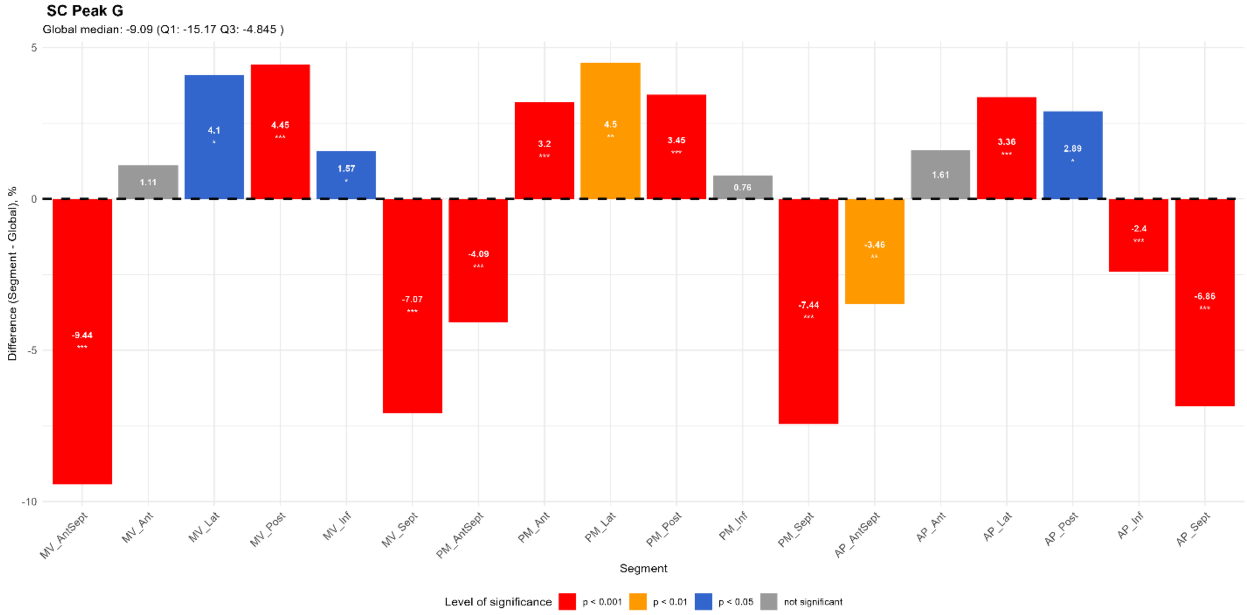

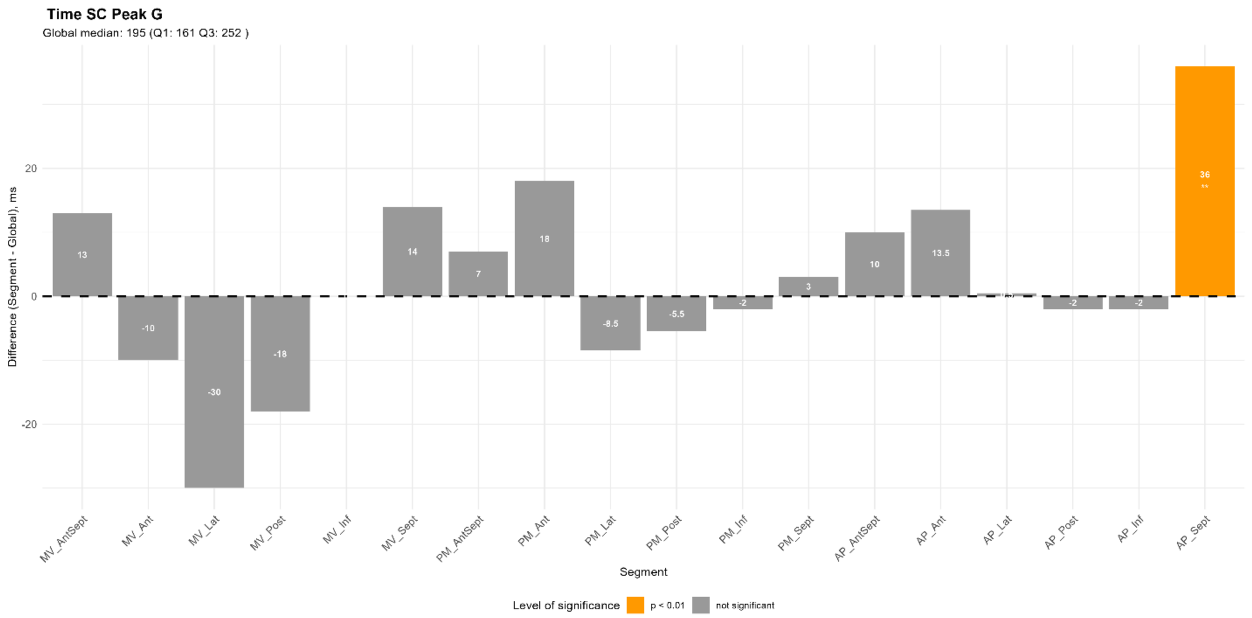
Time SC Peak G
Global median: 195 (Q1: 161 Q3: 252)
Difference (Segment - Global), ms
Segment
Level of significance
p < 0.01
not significant

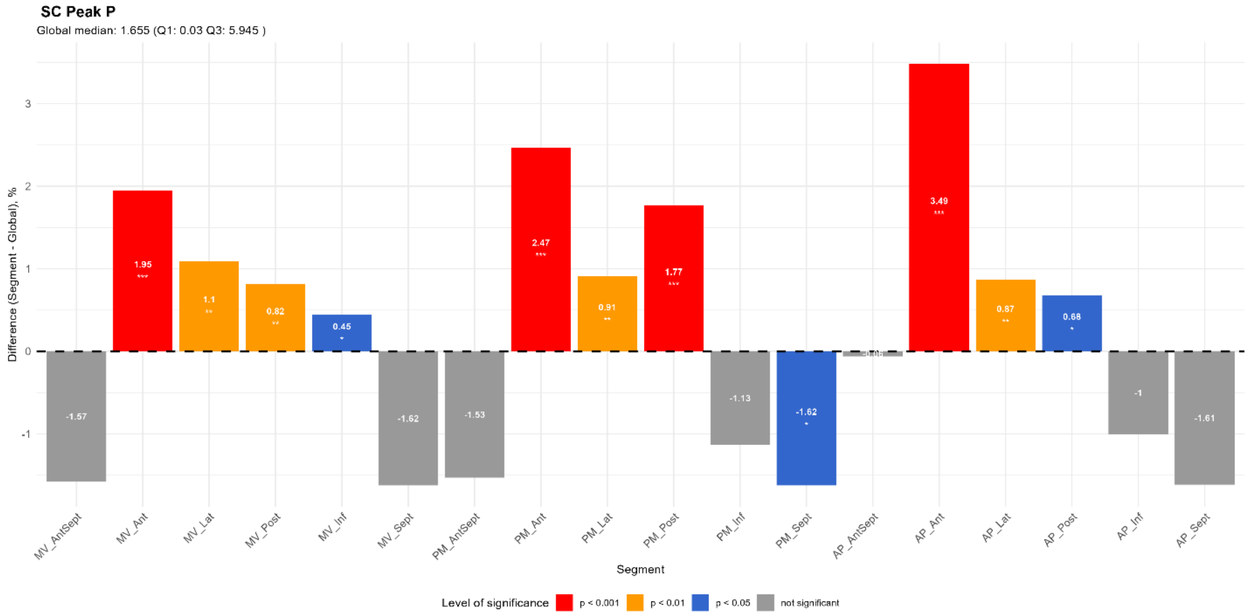
SC Peak P
Global median: 1.655 (Q1: 0.03 Q3: 5.945)
Difference (Segment - Global), %
Segment
Level of significance
p < 0.001
p < 0.01
p < 0.05
not significant

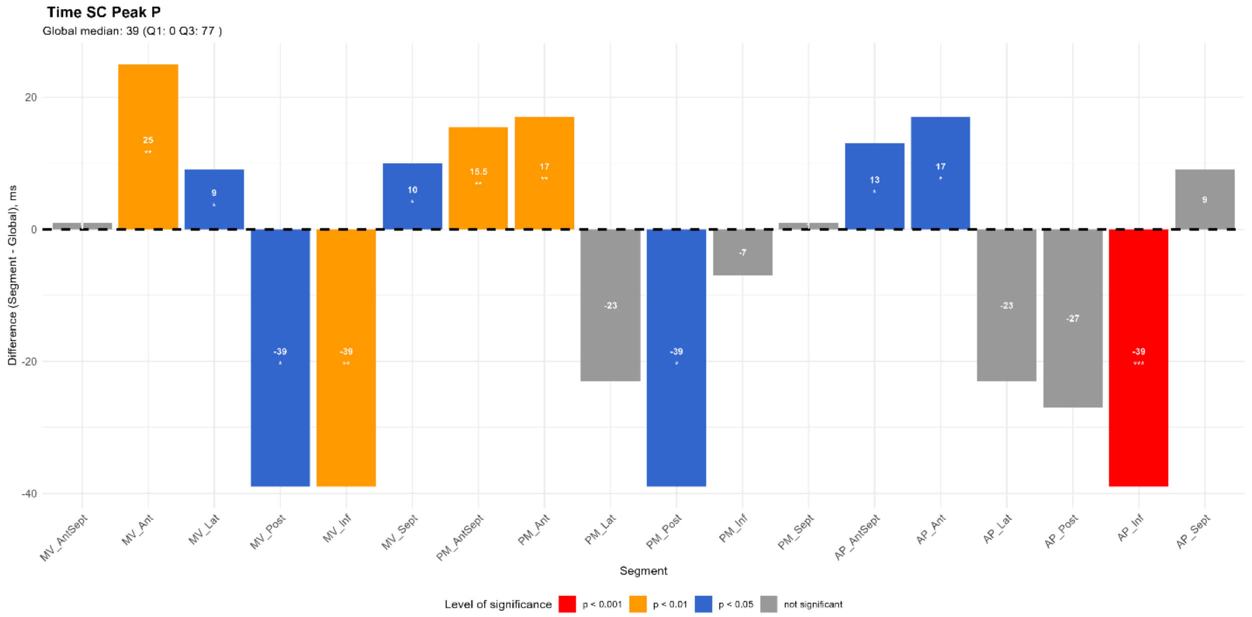
Time SC Peak P
Global median: 39 (Q1: 0 Q3: 77)
Difference (Segment - Global), ms
Segment
Level of significance
p < 0.001
p < 0.01
p < 0.05
not significant

C. Radial strain

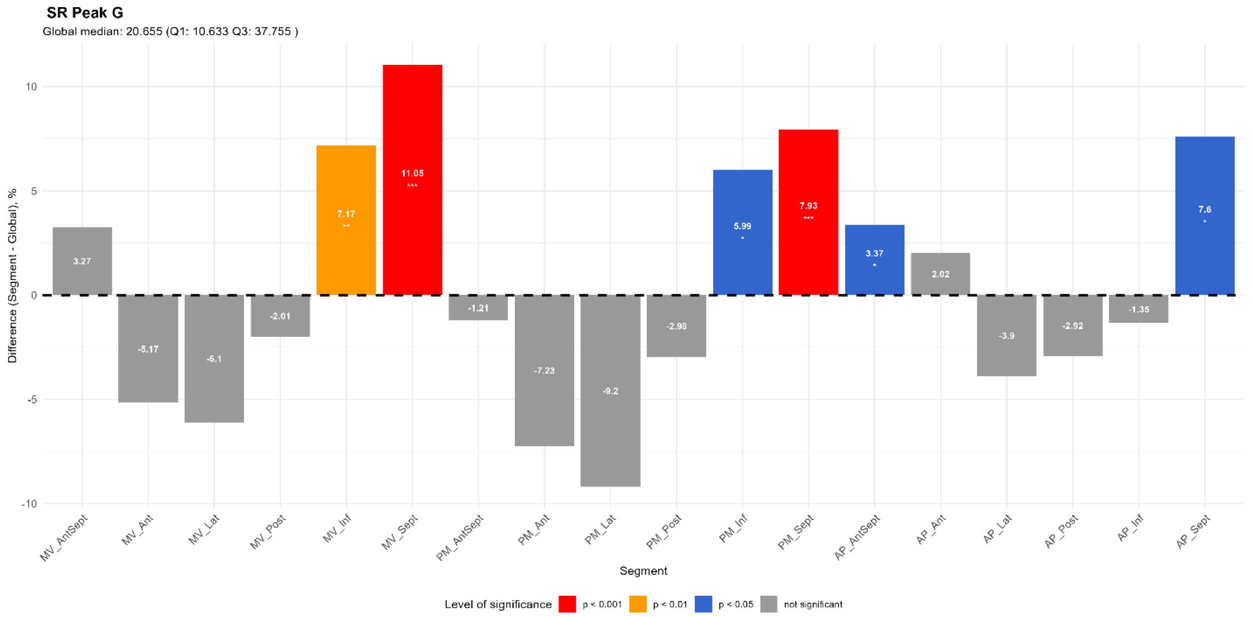

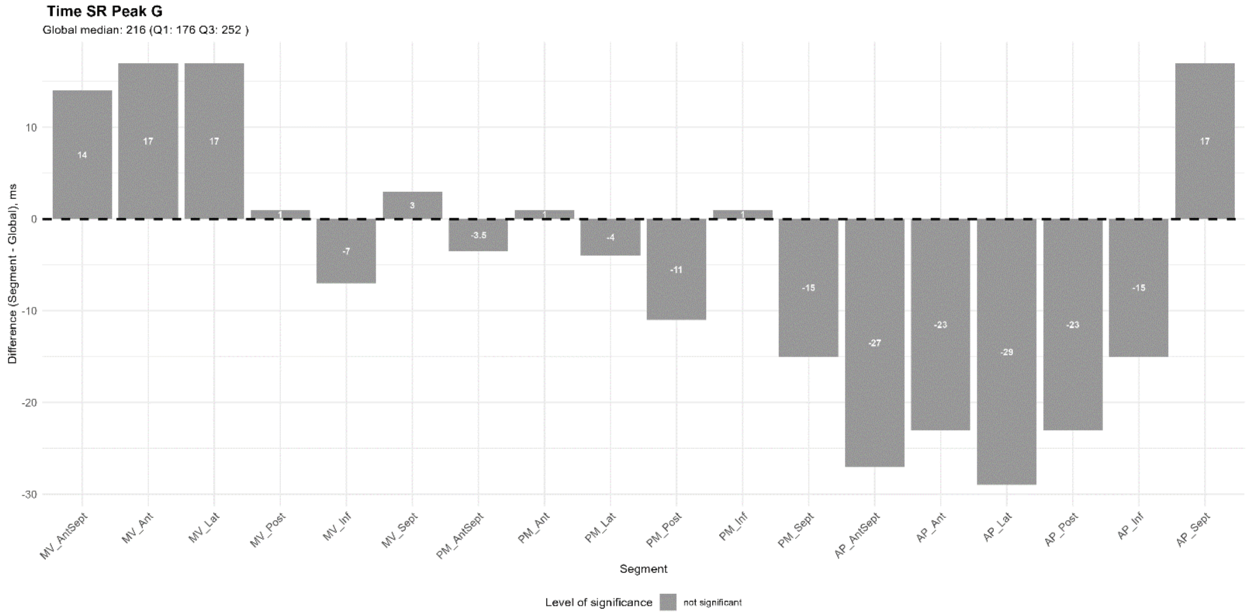

Figure S3. Heatmaps of the significance of differences in segmental parameters of the strain between LV segments.

A. Longitudinal strain

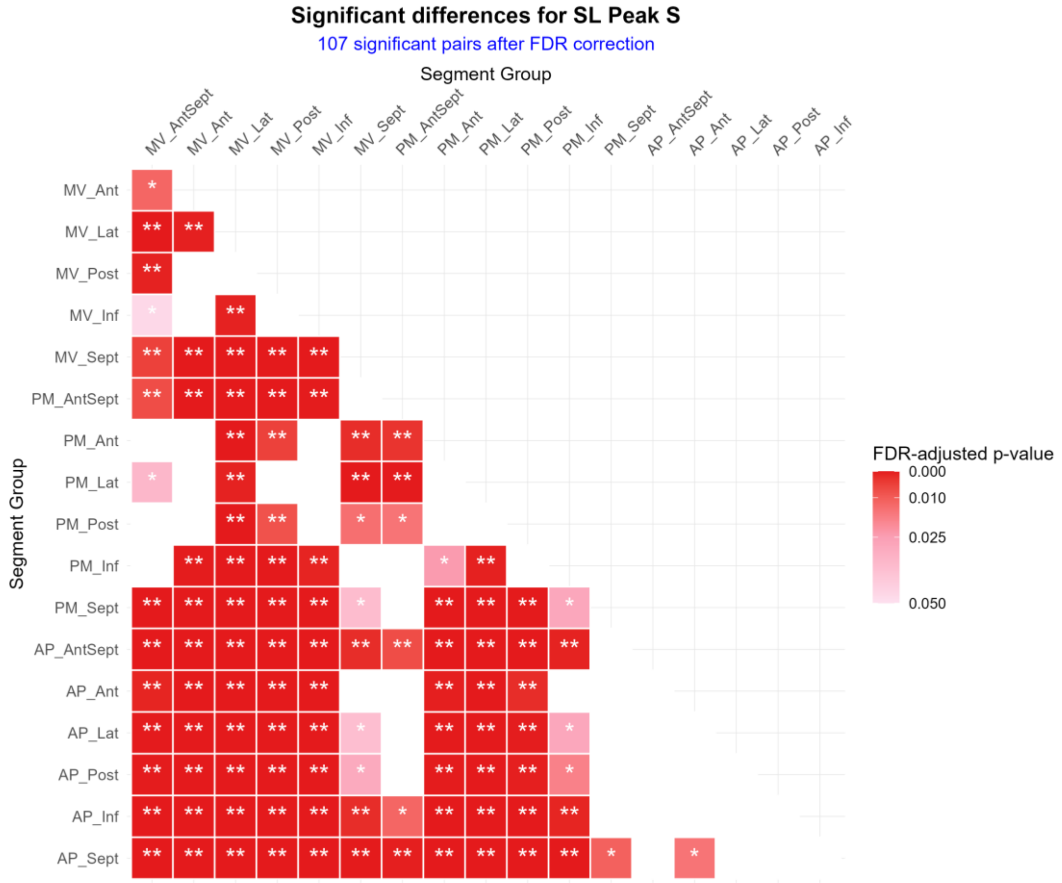

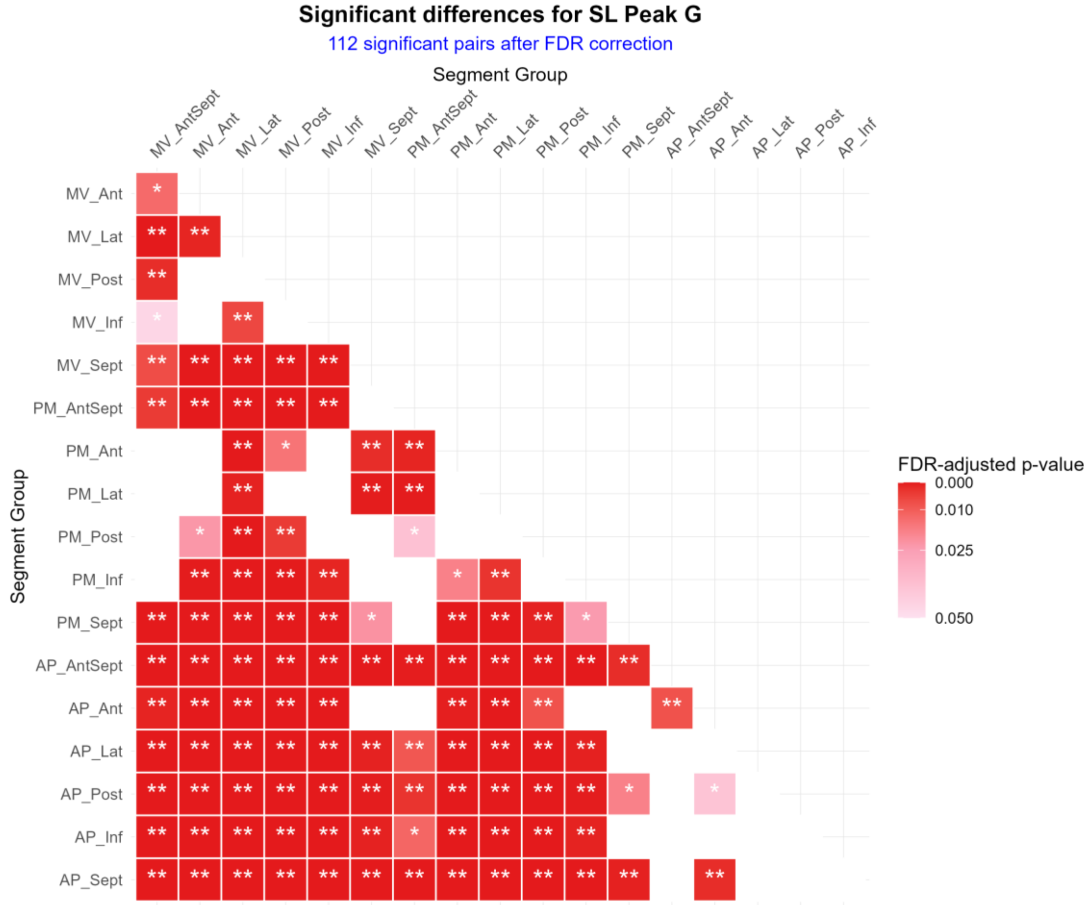

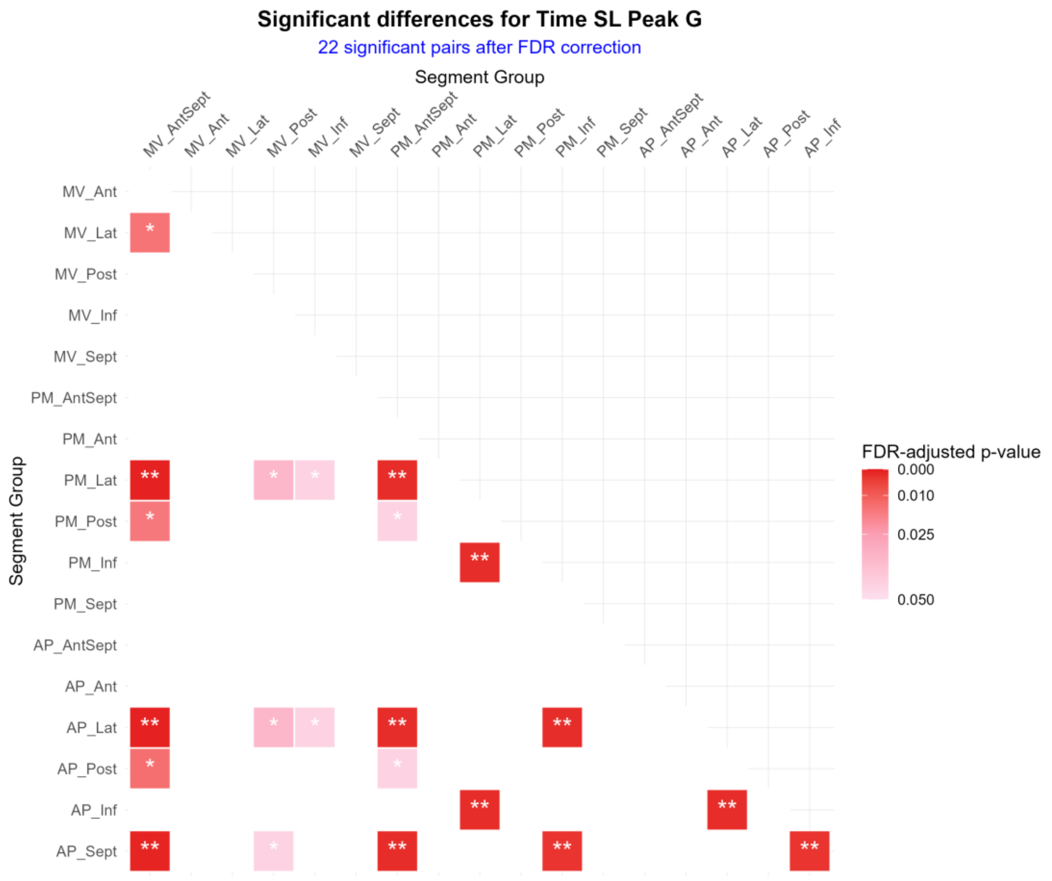

Significant differences for Time SL Peak G
22 significant pairs after FDR correction
Segment Group
MV_AntSept
MV_Ant
MV_Lat
MV_Post
MV_Inf
MV_Sept
PM_AntSept
PM_Ant
PM_Lat
PM_Post
PM_Inf
PM_Sept
AP_AntSept
AP_Ant
AP_Lat
AP_Post
AP_Inf
AP_Sept
Segment Group
FDR-adjusted p-value
0.000
0.010
0.025
0.050

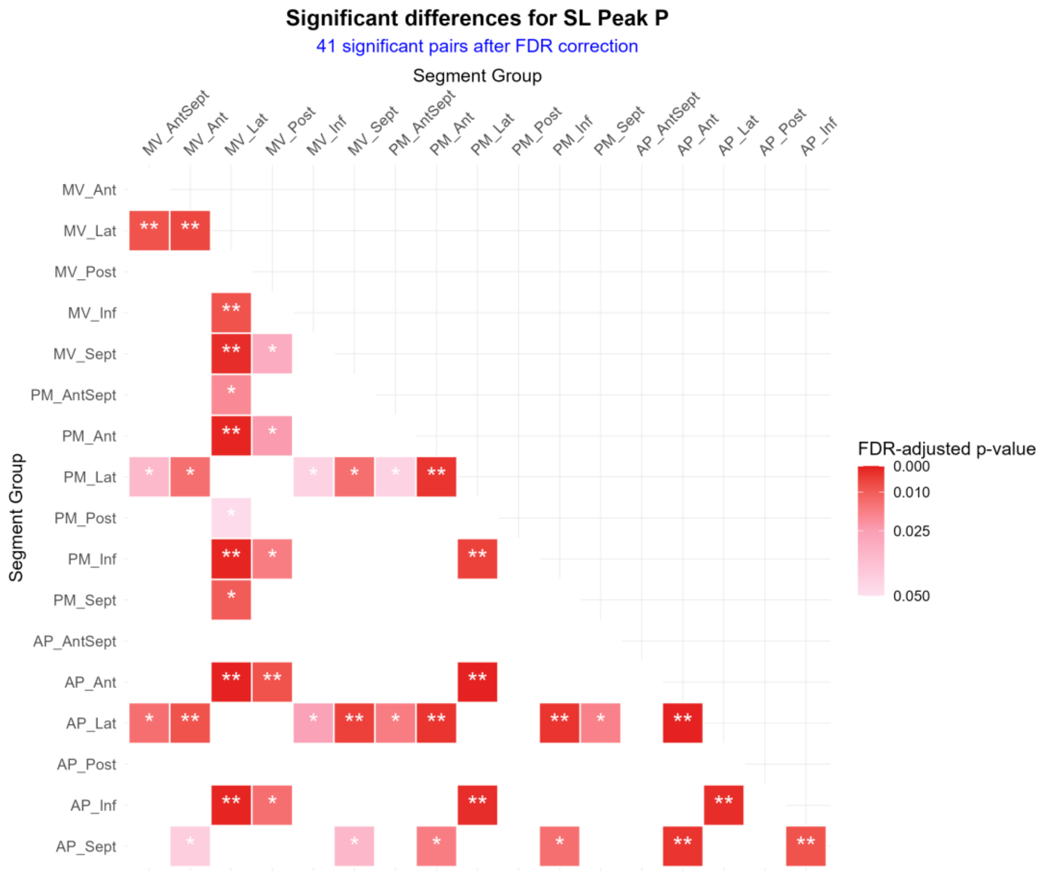

Significant differences for SL Peak P
41 significant pairs after FDR correction
Segment Group
MV_AntSept
MV_Ant
MV_Lat
MV_Post
MV_Inf
MV_Sept
PM_AntSept
PM_Ant
PM_Lat
PM_Post
PM_Inf
PM_Sept
AP_AntSept
AP_Ant
AP_Lat
AP_Post
AP_Inf
AP_Sept
Segment Group
FDR-adjusted p-value
0.000
0.010
0.025
0.050

B. Circumferential strain

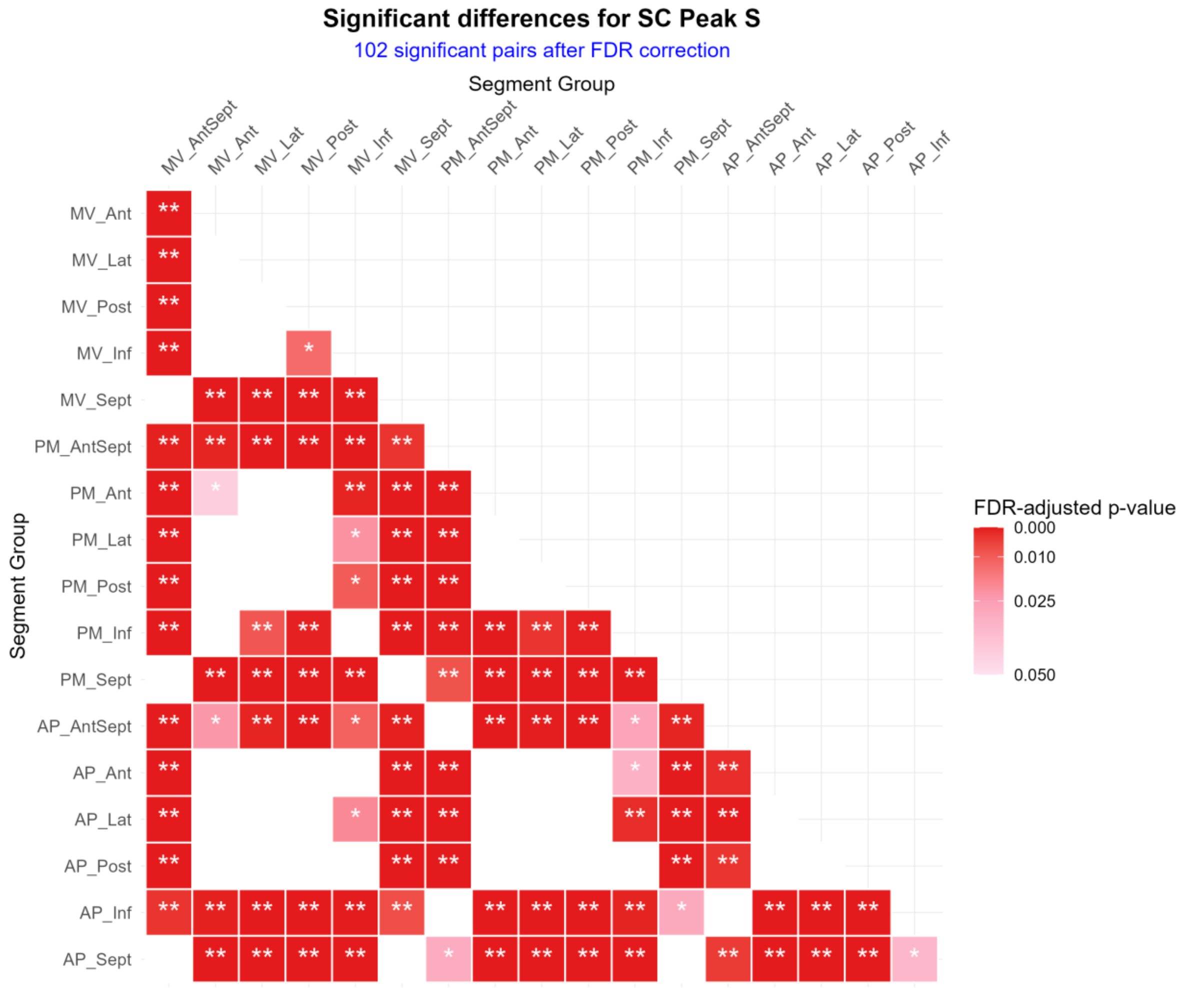

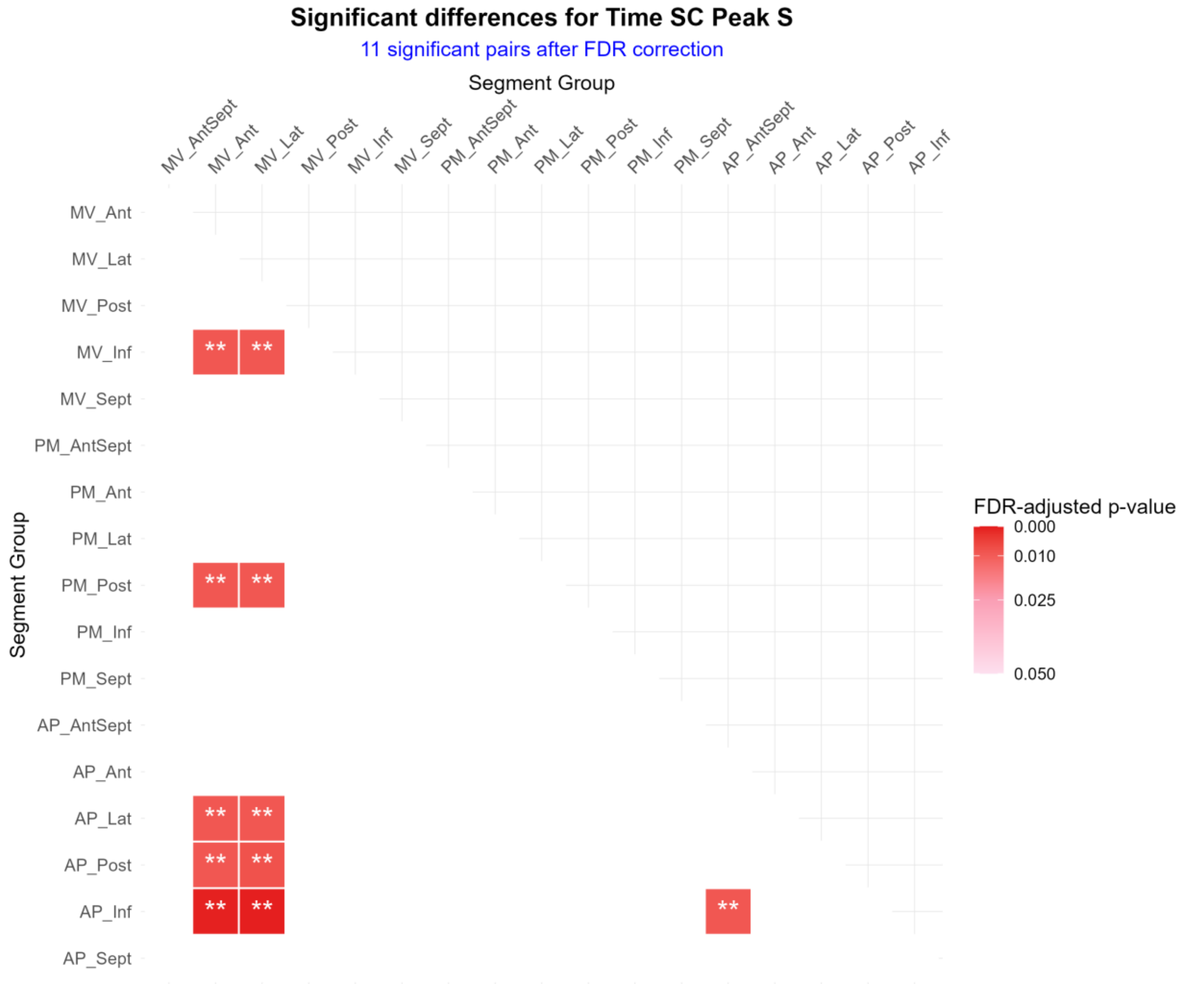

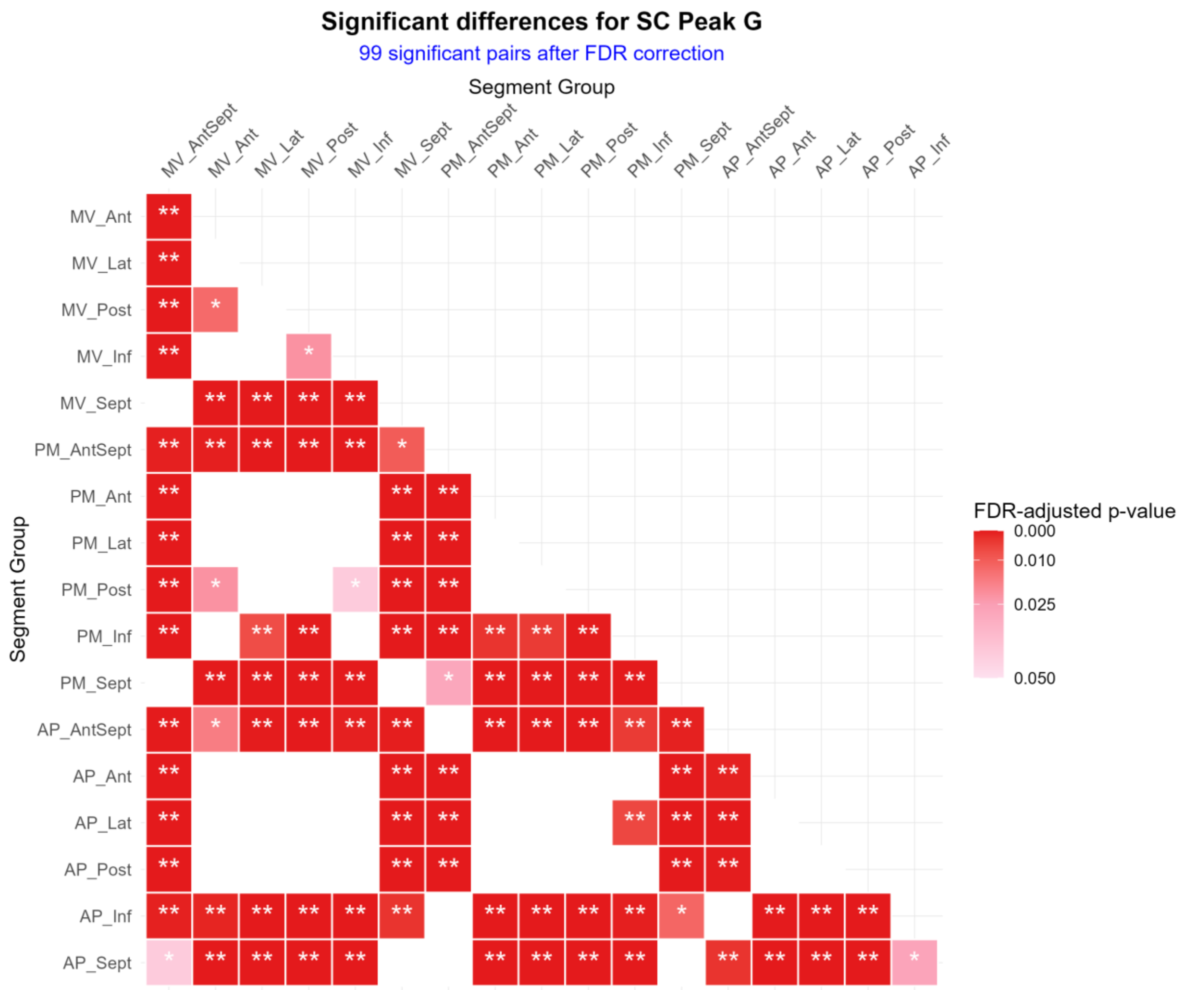
Significant differences for SC Peak G
99 significant pairs after FDR correction
Segment Group
MV_AntSept
MV_Ant
MV_Lat
MV_Post
MV_Inf
MV_Sept
PM_AntSept
PM_Ant
PM_Lat
PM_Post
PM_Inf
PM_Sept
AP_AntSept
AP_Ant
AP_Lat
AP_Post
AP_Inf
AP_Sept
FDR-adjusted p-value
0.000
0.010
0.025
0.050

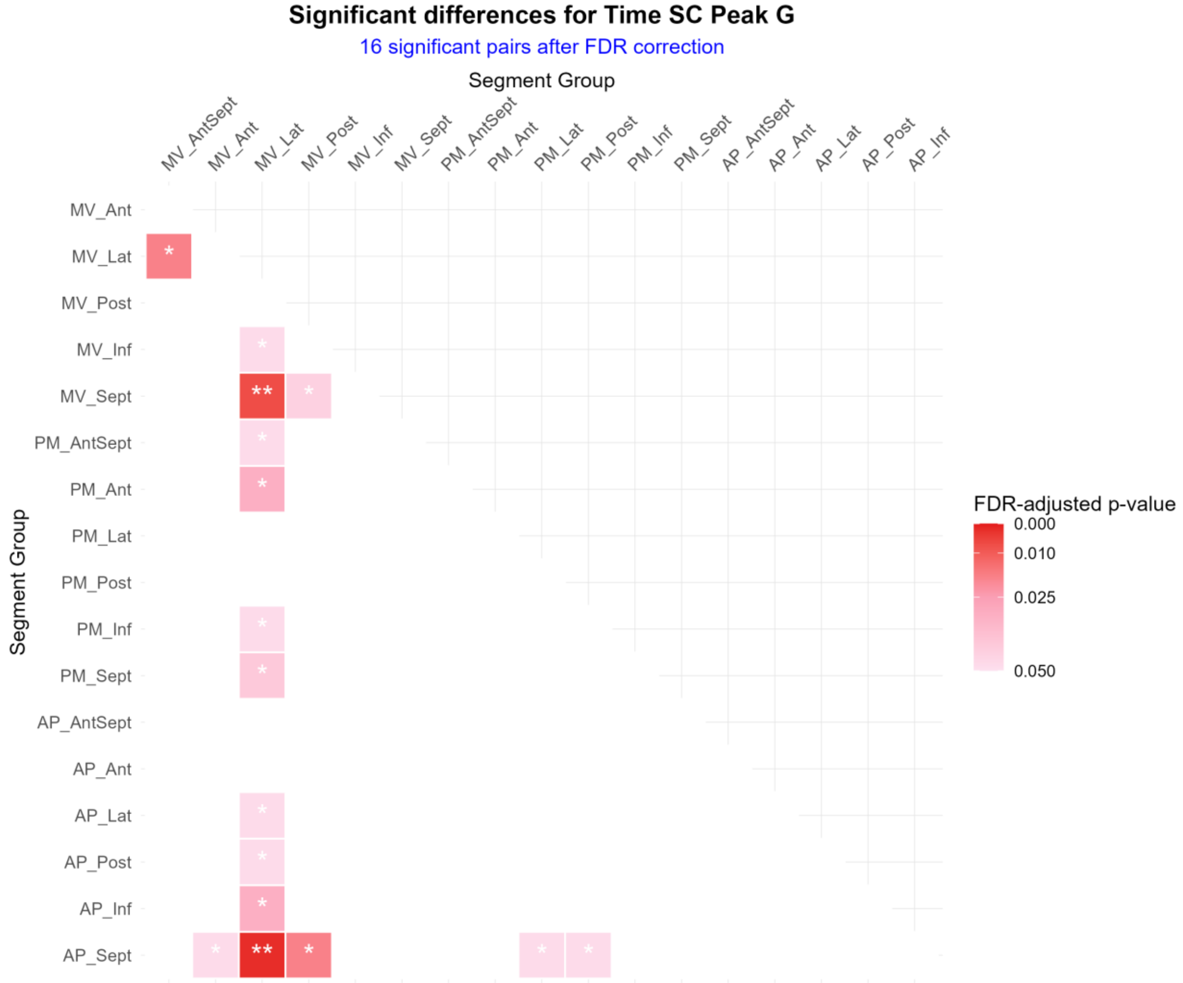
Significant differences for Time SC Peak G
16 significant pairs after FDR correction
Segment Group
MV_AntSept
MV_Ant
MV_Lat
MV_Post
MV_Inf
MV_Sept
PM_AntSept
PM_Ant
PM_Lat
PM_Post
PM_Inf
PM_Sept
AP_AntSept
AP_Ant
AP_Lat
AP_Post
AP_Inf
AP_Sept
FDR-adjusted p-value
0.000
0.010
0.025
0.050

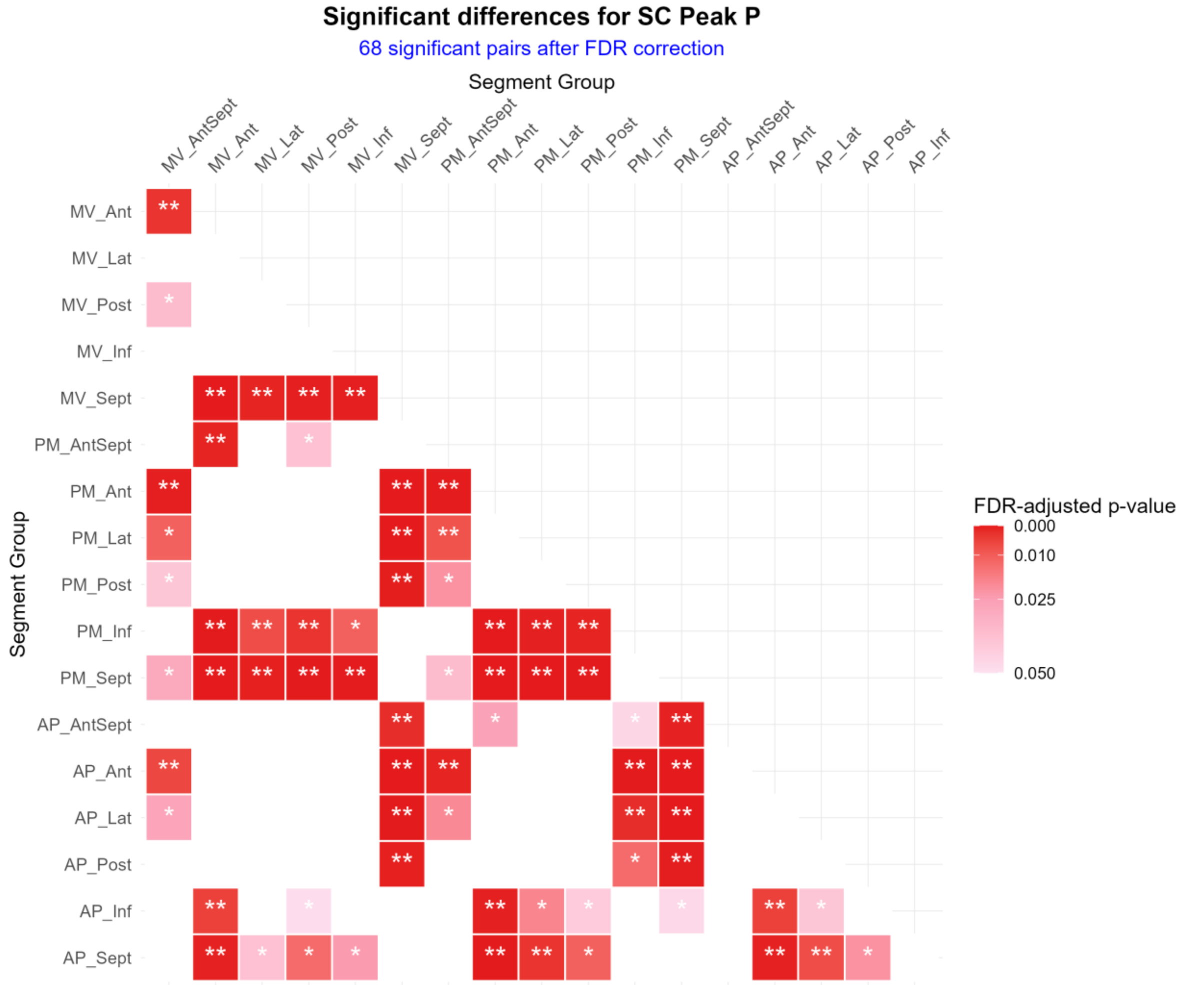

Significant differences for SC Peak P
68 significant pairs after FDR correction
Segment Group
MV_AntSept
MV_Ant
MV_Lat
MV_Post
MV_Inf
MV_Sept
PM_AntSept
PM_Ant
PM_Lat
PM_Post
PM_Inf
PM_Sept
AP_AntSept
AP_Ant
AP_Lat
AP_Post
AP_Inf
AP_Sept
Segment Group
FDR-adjusted p-value
0.000
0.010
0.025
0.050

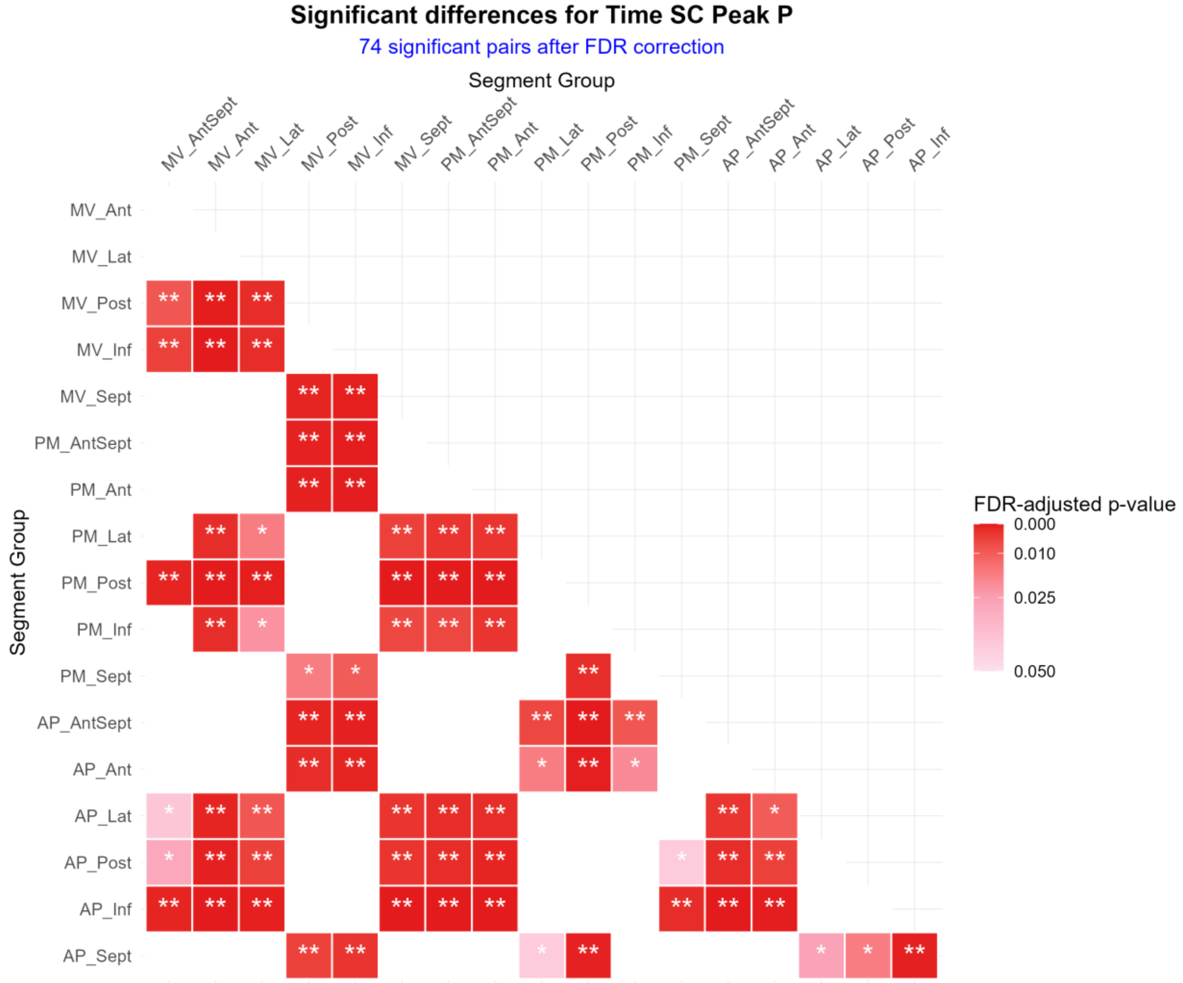

Significant differences for Time SC Peak P
74 significant pairs after FDR correction
Segment Group
MV_AntSept
MV_Ant
MV_Lat
MV_Post
MV_Inf
MV_Sept
PM_AntSept
PM_Ant
PM_Lat
PM_Post
PM_Inf
PM_Sept
AP_AntSept
AP_Ant
AP_Lat
AP_Post
AP_Inf
AP_Sept
Segment Group
FDR-adjusted p-value
0.000
0.010
0.025
0.050

C. Radial strain

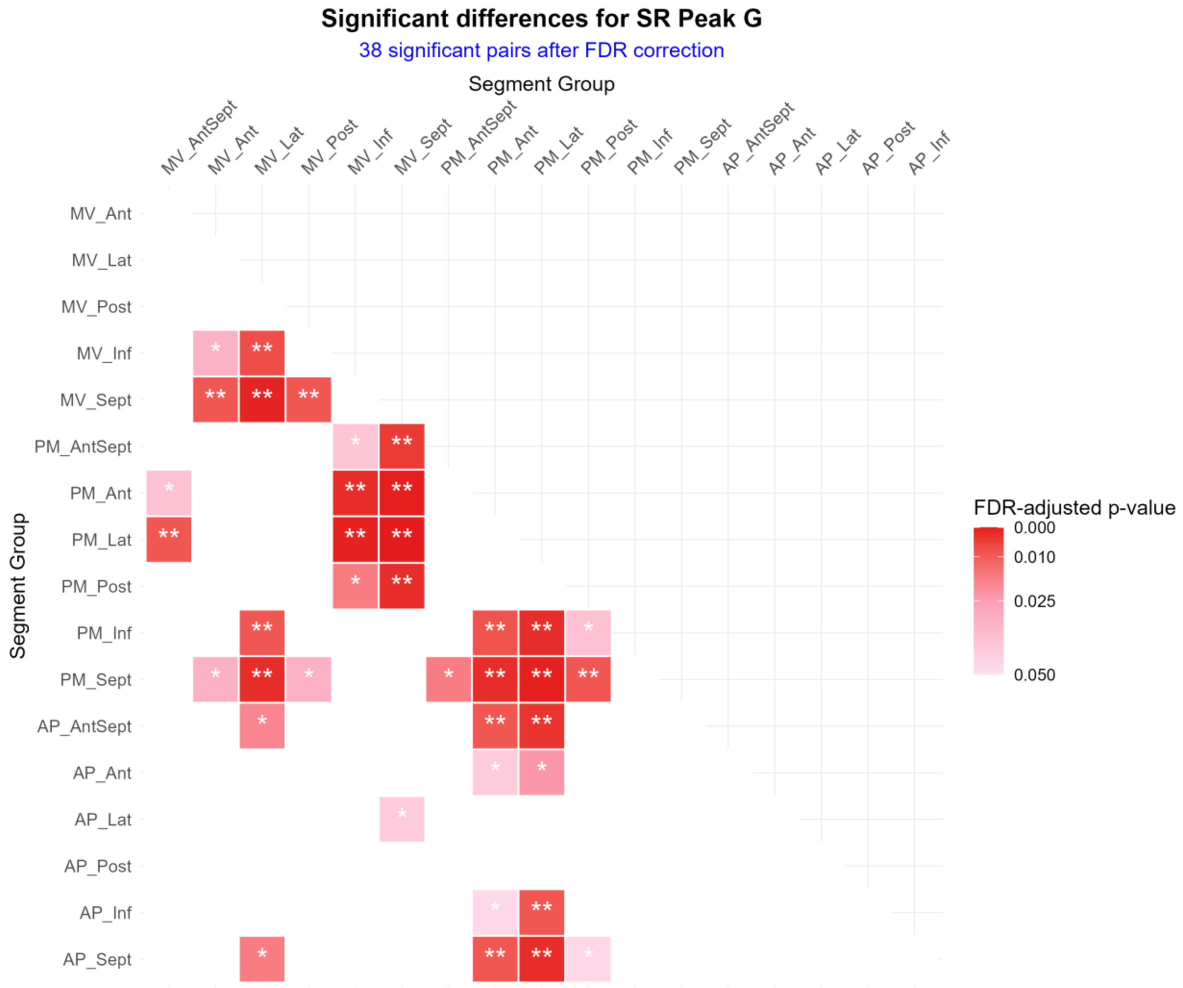

Supplement: Supplementary file 1 [file pediatrrep-17-00126-s001.zip › pediatrrep-3938451-supplementary.pdf]
